# Silibinins and curcumin as promising ligands against mutant cystic fibrosis transmembrane regulator protein

**DOI:** 10.1186/s13568-024-01742-z

**Published:** 2024-07-23

**Authors:** Areeba Akram, Azra Sakhawat, Muhammad Usman Ghani, Muhammad Umer Khan, Raima Rehman, Qurban Ali, Peng Jin-liang, Daoud Ali

**Affiliations:** 1 Precision Genomics Research Lab, Centre for Applied Molecular Biology, University of the Punjab, Lahore, Pakistan; 2 Institute of Molecular Biology and Biotechnology, The University of Lahore, Lahore, Pakistan; 3 Centre of Excellence in Molecular Biology, University of the Punjab, Lahore, Pakistan; 4 Department of Plant Breeding and Genetics, Faculty of Agricultural Sciences, University of the Punjab, Lahore, Pakistan; 5 Department of Emergency, The Affiliated Ganzhou Hospital of Nanchang University, Ganzhou, 341000 Jiangxi People’s Republic of China; 6 Department of Zoology, College of Science, King Saud University, PO Box 2455, Riyadh, 11451 Saudi Arabia

**Keywords:** Cystic fibrosis, Trikafta, Mutation, Molecular docking molecular simulation, ADMET

## Abstract

Cystic Fibrosis Transmembrane Regulator (CFTR) is a significant protein that is responsible for the movement of ions across cell membranes. The cystic fibrosis (CF) occur due to the mutations in the *CFTR* gene as it produces the dysfunctional CFTR protein. The sequence of CFTR protein as a target structure was retrieved from UniProt and PDB database. The ligands selection was performed through virtual screening and top 3 ligands choose out of 65 ligands silibinins, curcumin, demethoxycurcumin were selected with a reference drug Trikafta (R*). According to docking, ADMET analyses, the natural ligands (Silibinins and Curcumin) displayed best binding energy, pharmacokinetic and free toxicity than other natural compounds and reference drug (R*). An MD simulation for 200 ns was also established to ensure that natural ligands (Silibinins and Curcumin) attached to the target protein favorably and dynamically, and that protein–ligand complex stability was maintained. It is concluded that silibinins and curcumins have a better capacity to decrease the effect of mutant CFTR protein through improved trafficking and the restoration of original function. In conclusion, in silico studies demonstrate the potential of silibinins and curcumin as therapeutic agents for cystic fibrosis, particularly for the D614G mutated protein. Their ability to increase CFTR function while reducing cellular stress and inflammation, together with their favorable safety profile and accessibility could make them valuable additions to cystic fibrosis treatment options. Further experimental and clinical validation will be required to fully realize their potential and include them into effective therapy regimens.

**Supplementary Information:**

The online version contains supplementary material available at 10.1186/s13568-024-01742-z.

## Introduction

Cystic fibrosis (CF) is the most frequent life-threatening, monogenic illness among Caucasians. Its frequency in the European population is roughly one in every 2500 babies, and one in every 3500 newborns in the US (Ashraf et al. 2023a, b; Sipione et al. 2023). Cystic fibrosis is caused by a mutation in a specific gene known as cystic fibrosis transmembrane conductance regulator. The CFTR gene is located on the long arm of chromosome 7, at q31. The size of the gene is approximately 250 kb (Ramananda et al. 2024). This gene comprises 27 exons and encodes a mature mRNA with a size of 6.5 kb and 1480 amino acids (Bradbury et al. 2020). The CFTR protein is made from the instructions on the *CFTR* gene, which are copied in the mRNA and then translated into the protein in ribosomes. This CFTR protein is processed in the endoplasmic reticulum before it moves to its destination in the cell (Mitrick 2023).

Cystic fibrosis affects multiple organs, causing problems like the accumulation of thick mucus around the airways, blocking the airway, and leading to chronic lung infection, inflammation, and bronchiectasis. It also affects the pancreatic ducts leading to pancreatic insufficiency, bowel obstruction in newborns, and a distinctive high salt sweat that helps in the diagnosis of the disease. Cure for cystic fibrosis is still limited; only improvements in patient care and symptom management over the past decades can increase the life expectancy of CF patients. In 1950 the life expectancy was around 1 year and now it has extended up to 40 years (Flume et al. 2009). Nowadays different modulation therapies are used for the treatment of CF depending on the type of mutation (Lopes-Pacheco et al. 2021).

CFTR mutations are categorized into six different classes based on their impact on protein functionality. In a typical scenario, the CFTR protein is synthesized and migrates to the cell surface, where it plays a critical role in facilitating the passage of chloride and water across the cell membrane (Ashraf et al. 2023a, b; Shan et al. 2024). In Class I and II mutations fall under the category where the CFTR protein is produced, but it adopts a misfolded conformation, preventing it from properly reaching the cell surface. This condition is known as a trafficking defect. Class III mutations result in the production of CFTR protein that successfully reaches the cell surface, but fails to open the channel gate, resulting in defective channel regulation. In class IV mutations normal CFTR protein is formed and it does reach the cell surface but exhibits reduced channel functionality, causing decreased channel conductance. In Class V mutations, everything seems to operate smoothly, but insufficient quantities of CFTR protein are produced to fulfill its normal function, a condition termed reduced synthesis of CFTR. Finally, Class VI mutations involve the production of an adequate quantity of CFTR protein that fails to function effectively at the channel level, described as decreased CFTR stability (Cutting 2005; Veit et al. 2016). For example, extensively studied mutation F508del is categorized as a class II mutation. Interestingly it displays the characteristics of class III and IV defects as well. This observation finds validation in clinical settings where combination modulator therapies, aim to address the various mutation classes. It is more advantageous to rather than relying on a single pharmaceutical agent. Consequently, medications capable of rectifying any CFTR defect hold potential benefits for many mutation classes (Southern et al. 2020).

The natural compounds Curcumin, Genistein, and Resveratrol have all shown efficacy in this area; however, coumarins, quercetin, and other herbal drugs have also been shown to improve transporter function, transmembrane conductivity, and overall channel activity. Additional in vitro and in vivo research on mutant CFTR should be performed to unequivocally establish the mechanism by which phytochemicals modify transmembrane channel function/activity, as the results of the studies assessed in this review are highly heterogeneous and inconsistent. The therapeutic effects of therapeutic phytochemicals on the symptoms found in CF patients in order to reduce mortality and morbidity (Abbas et al. 2021; Baharara et al. 2023; Awan et al. 2024; Sheema et al. 2024; Ullah et al. 2024).

The natural coumarin compounds capable of correcting the faulty DF508-CFTR chloride channel gating by screening a collection of 386 single natural compounds from Chinese medicinal herbs. The iodide inflow assay was used in Fischer rat thyroid epithelial cells coexpressing DF508-CFTR and an iodide-sensitive fluorescent indicator (YFP-H148Q/I152L). The natural coumarin DF508-CFTR activators could be a novel class of natural lead molecules for the development of pharmacological treatments for CF caused by the DF508 mutation (Xu et al. 2008).

Many missense variants affect the CFTR protein structure and function. One such variant is D614G, in which aspartic acid at position 614 changes to glycine. In this study, we used various bioinformatics tools to identify missense variants and their consequences. These tools have been broadly used in previous studies and have played a role in the screening of mutations in numerous research experiments. Each tool used in our study provides a score value for identifying harmful mutations.

The study aimed to estimate the effect of the CFTR missense variant specifically D614G and to get deep insight into the mechanism of action of the drugs and natural compounds. We used in silico analysis, molecular docking, MD simulation to determine whether natural drugs bind to the mutated structure of CFTR and restore its normal function.

## Methodology

The variant D614G of CFTR protein is evaluated using various bioinformatics tools and methodology see (supplementary Fig. 1).

### Data retrieval

The amino acid sequence for wild-type cystic fibrosis transmembrane regulator (CFTR) protein was obtained from the UniProt database. CFTR human protein UniProt Id is P13569, (accessed on 5th Jan 2024). For the study of cystic fibrosis variants, we took information from the ClinVar database accessible. This is a freely accessible source, which gathers information on different variants, their clinical significance, and clinical phenotypes. These bioinformatics tools classified the variant as benign, likely benign, pathogenic, or nonpathogenic.

### Functional analysis

#### Screening of deleterious single nucleotide substitutions

In this research study, our focus was solely directed towards the examination of variants, resulting in amino acid substitutions. Consequently, we took our variant from 779 missense variants associated with the *CFTR* gene, data present in the CFTR 2(https://cftr2.org/)- the database was last updated on April 7, 2023. It classified the variant as CF-causing, non-CF causing, and unknown significant. The following computational tool did the screening.

#### Predicting functional effect of mutation on CFTR protein

##### Sorting of intolerant from tolerant

SIFT (sorting intolerant from tolerant) is a freely downloadable utility. This tool is intended to evaluate the effect of amino acid changes on protein function and to compare both the physical properties and sequence homologies of protein sequences. It informs us about the mutation and the prospective changes in the phenotype. It also distinguishes which mutation is harmful and neutral and which mutation can significantly affect the protein function (Adzhubei et al. 2010). The output value for SIFT ranges from “0” to “1” where zero signifies the damaging effect and one signifies the neutrality.

#### Poly-Phen2

The functional consequences of the mutation on CFTR protein are evaluated by using the two in silico tools i.e., Poly-Phen2. It is a freely available online tool accessible (Choi and Chan 2015) used to check the effect of missense mutation damaging effect. Users often submit the protein’s FASTA sequence as well as the precise amino acid substitution site when using the PolyPhen-2 tool. The amino acid residue in question is entered into the opposite boxes, with wild-type residues in AA1 and mutant residues in AA2.

#### Protein stability analysis

The missense mutation can impact the protein's stability, which in turn affects its function. We used various bioinformatics methods to evaluate and analyze protein stability changes.

#### I-Mutant 2.0

I- Mutant 2.0 is an easily accessible web server utility (Capriotti et al. 2005) based on the support vector machine (SVM). Its objective is to determine the impact of mutations on protein stability. This concentrates on the delta G value caused by the missense mutation. The Delta G value is used to predict protein stability; a negative number indicates improved stability, whereas a positive value indicates destabilization. Users can estimate the effect of protein stability changes by entering the CFTR protein sequence, the site of the mutation, and a description of the new amino acid residue.

#### MUpro

MUpro is an internet server program developed to forecast the changes in protein stability caused by the mutation (Cheng et al. 2006). SVMs (support vector machines) are machine learning methods used to assess protein stability. This tool likewise uses an SVM score to forecast the protein alterations induced by a single amino acid mutation. In the input field, the user enters the wild type and mutant residues of amino acids, as well as the CFTR protein sequence. Protein model performance is measured using criteria such as accuracy, precision, and correlation efficiency.

#### DDGun

Single amino acid substitution affects protein stability. To these numerous techniques have been developed to measure the number of Gibbs free energy while comparing the wild type and variant type. This job is done by using the DDGun (https://folding.biofold.org/ddgun/) freely accessible software (Pejaver et al. 2020).

#### DUET

The effect of D614G mutation on protein stability is also determined using the DUET (https://bio.tools/duet) freely accessible bioinformatics tool (Pires et al. 2014). Delta G value indicates how specific mutations affect the protein stability. A positive value indicates that the protein structure will be destabilizing due to the substitution of that specific amino acid.

#### SDM

Site detector mutator (http://www.sdmtoolbox.org/) is a computational tool used to predict the effect of single amino acid substitution on protein stability (Worth et al. 2011). Input the detail of mutation of interest into the SDM model and this model will predict the change in the free energy associated with the mutation. A positive value indicates a decrease in stability while a negative value indicates increased stability.

#### Maestro web

Maestro web is a computational tool (https://pbwww.services.came.sbg.ac.at/maestro/web) (Laimer et al. 2016). Entered the pdb id and uploaded the structure of CFTR protein. Select the appropriate method either algorithm or energy-based method. We focused on the change in energy method for D614G variant.

#### DynaMut

It is a freely accessible tool. Upload the wild type of structure of CFTR protein enter the mutation detail in the given box and click on run prediction. Results are displayed in the four different tabs. First tab will show the trajectory movement; the second tab will indicate the animated plot that describes the motion of the molecule. Visual representations of deformation energy and atomic fluctuation are shown in the third tab. Finally, the last tab showed the correlation between the residue movement of wild and mutated type proteins (Sierra and Brini 2024).

#### GOR IV

The GOR IV method (https://npsa-pbil.ibcp.fr/cgi-bin/npsa_automat.pl?page=/NPSA/npsa_gor 4.html) analyzes the protein sequence to predict how many alpha helixes, beta sheets and random coil are present in the secondary structure of it. It utilizes an information theory-based method which is used for the prediction of the secondary structure of protein. In this tool sequence of mutated CFTR proteins is submitted and it will generate the secondary structure it (Deléage 2017).

### Predicting the structural alignment of protein

TM- align is a protein structural comparison technique that works independently of sequencing. The wild and mutant types of CFTR are uploaded in the two boxes before running the TM-align.

### Predicting the effect of single nucleotide substitution on the structure and function of protein

Bioinformatics tools are critical for assessing the mutation's detrimental effect. These methods are intended to estimate the statistical impact of mutations on protein structure and function. MutPred 2.0, which is available at (http://mutpred.mutdb.org/), not only identifies the likely mechanism of a disease-causing protein but also provides a pathogenicity score. It retrieved the FASTA sequence of the CFTR protein, which included the amino acid substitution. Scores greater than 0.5 suggest that the specific mutation has a harmful effect (Li et al. 2009).

### Predicting the physiochemical properties

Protparam (https://web.expasy.org/protparam/) is a freely available tool for determining the physical and chemical properties of a protein. Enter the protein's accession number into the box or paste the amino acid sequence of CFTR (Gasteiger et al. 2003).

### Presence in functional domains

Pfam accessible at (http://pfam.xfam.org/) is an extensive database used for getting information about the domains of the CFTR protein. Researchers typically input the protein ID into the Pfam server to reveal the functional domain within the protein (Mistry et al. 2021).

### PTM analysis

Posttranslational alteration involves amino acid alterations caused by the addition of various side chains or functional groups to the CFTR protein. PTM affects protein structure and function both directly and indirectly. Mutations at PTM regions can change protein function by influencing regulatory signals or stability. Any mutation on the side chain can cause illness. This study used the MusiteDeep server to find PTM locations in the CFTR protein. The server was tasked with detecting any PTMs in the CFTR protein using the FASTA sequence acquired from NCBI (Wang et al. 2020).

### Analyzing the impact of single nucleotide substitutions on protein properties

A server called HOPE (https://www3.cmbi.umcn.nl/hope/input/) is used to investigate the effect of mutation on protein attributes such as size, charge, hydrophobicity, structure, and so on. The protein sequence or accession number was supplied along with the change in amino acid to check the structural consequences of the mutation (Venselaar et al. 2010).

### Prediction of secondary structure

The secondary structure of CFTR protein is determined by using Ramachandran plot, ERRAT, and verify 3D. For secondary structure validation of CFTR standard and mutant PDB structure is uploaded and then run the programs.

### Homology modelling

The homology modeling of CFTR protein was carried out using PyMol software (https://pymol.org/2/) and the Swiss model (https://swissmodel.expasy.org/) (Schwede et al. 2003). The structural template for this modeling was obtained from a protein data bank (PDB) with PDB ID 6MSM. Open PyMol and load your protein. Go to Wizard select mutagenesis and replace the Aspartic acid at 614 positions with Glycine.

### Virtual screening of ligands for CFTR

We selected these natural compounds because of their anti-inflammatory and antioxidant properties, guided by the extensive literature review. PubChem was used for the virtual screening of natural compounds. The following ligands are used in this article; curcumin (969516) compound CID, demethoxycurcumin compound CID (5469424), and silibinins compounds CID (31,553).

### Predicting the effect of *CFTR* mutant complex on binding affinity

#### Protein preparation

PyMOL was used to examine the 3D crystal structure of the CFTR (D614G) mutant protein to predict its function and stability. The protein was created and sent to Auto Dock Vina for molecular docking, where the missing atoms were examined, reconstituted, and Kollman's charges were applied. The polar hydrogen was likewise dissolved, and the finished product was stored as a PDBQT file.

#### Ligands preparation

The ligands were exported into the PyMOL and converted into PDB format. The three-dimensional structures of the compounds were taken from PubChem and exported to PyMOL to convert into PDB format. Subsequently, the enhanced ligands were saved in the PDBQT file format after being exported to AutoDock Vina, where they underwent further preparation, including the removal of heteroatoms from the ligand preparation, energy minimization, and the addition of hydrogen atoms.

#### Analyzing the toxicity profile of natural ligands

Swiss ADME is a computational technique for predicting the ADME parameters, pharmacokinetics, and drug similarity of both natural and synthesized drugs (Daina et al. 2017). Predicted were physically relevant characteristics and descriptors of pharmacological significance for the target ligands. The following properties were predicted by Swiss ADME: molar refractivity, topological polar surface area, number of hydrogen bond donors/number of hydrogen bond acceptors, skin permeation (log Kp), drug-likeness (Lipinski’s rule of five), cytochrome-P enzymes inhibition, GI absorption, BBB permeation, P-gp substrate, lipophilicity (logPo/w), and skin permeation.

#### Analyzing the MD simulation of natural ligands

Protein ligand interaction was studied by molecular docking and the best dock protein–ligand combination was simulated using the Desmond software, a molecular dynamics technique that makes use of set optimal potentials for liquid simulations (OPLS-2005) force field (Kumar et al. 2020). The system was prepared for simulation by setting the size and shape of the orthorhombic box at 10 Å × 10 Å × 10 Å and using a simple point charge (SPC) model as the solvent in the box with periodic boundary conditions. With buffer regions between the box sides and protein residues of 0.15 M NaCl 10 Å, the desired electrically neutral system for the simulation was built using the system builder tool. The steepest decline in short-term memory. The 200 ns molecular dynamics simulation was performed using the Schrödinger LLC Desmond software. The protein and ligand docking was carried out before the MD simulation, and it was an essential step in predicting the static view of the molecule's binding position in the protein's active site. In this case, the OPLS_2005 force field, an orthorhombic box at 300 K, and a pressure of 1 atm were used as the basis for the TIP3P solvent model. To neutralize the models and replicate physiological circumstances, 0.15 M sodium chloride and counter ions were employed, respectively.

#### MM-GBSA calculation

The MM/GBSA method was used to determine the binding free energy of the whole complex, which integrates the ideas of generalized Born surface area modeling with molecular mechanics. Using the Glide posture viewer file, the overall binding free energy was determined during the main MM-GBSA simulation. The energy minimization algorithms for the protein–ligand complexes (E-complex), free proteins (Eprotein), and free ligands (Eligand) were then run using these values. ΔGbind was used to compute the binding free energy (Genheden and Ryde 2015).

$$ \begin{aligned} \Delta {\text{Gblind}} = & {\text{E - complex}}\,{\mkern 1mu} {\text{(minimized)}} \\ & - {\text{E - ligand}}\,{\text{(minimized)}} \\ & - {\text{E - receptor}}\,{\text{(minimized)}} \\ \end{aligned} $$

A higher negative number shows greater binding since the MM-GBSA binding energies are approximations of the free energies of binding.

#### PCA analysis

To gain a better understanding of the movement of macromolecules, we examined the motion of molecular dynamics (MD) trajectories using Principle Component Analysis (PCA), a learning approach. The internal motions of the protein were the focus of this work. We computed the PCA and extracted valuable information from the simulation data using R script, and we performed the PCA using the R package Bio3D. The script played a major role in this study by helping us comprehend the molecular dynamics simulation on a deeper level.

## Results

### Prediction of functional consequences of single nucleotide substitutions

#### SIFT analysis

SIFT is a bioinformatics method that predicts how amino acid substitution affects protein function, prioritizing mutations to identify potentially harmful variants. The SIFT score of 0.05 distinguishes between harmful and tolerated amino acid substitutions; values below this level denote non-tolerant mutation, while scores above this level denote protein tolerance. The protein's capacity to function will be impacted by the D for G substitution at position 614. According to the SIFT study, its score is 0.05 indicating that this mutation has a damaging effect in (Supplementary Fig. 2) and Table S1 (Supplementary material tables).

#### PolyPhen-2

Human protein structure and function are affected by amino acid alterations, and PolyPhen-2 is a tool that forecasts these effects. A higher score indicates more harmful changes, whereas a lower score indicates benign ones. Its score runs from zero to one. The D614G mutation in the CFTR protein has a score of 1.000 (sensitivity: 0.00; specificity: 1.00), which suggests that it is probably damaging. A score close to 1 suggests a high probability of having a more damaging effect in Table S2 (Supplementary material tables).

#### PANTHER-PSEP

The PANTHER result categorized the variant as “probably damaging” and “possibly damaging” resulting in the consequence of mutation on protein. Variant D614G shows a value of 0.85 h means this mutation, is probably damaging in Table S2 (Supplementary material tables).

### Prediction of harmful single amino acid substitutions on protein stability

#### MUpro

To more fully comprehend genetic variants and protein functions, this machine learning method forecasts changes in protein stability brought on by single-site mutations. The delta G value obtained for mutation was less than zero i.e., − 1.734507 which signals towards decreased stability. The confidence score of the support vector machine method and the neural network was one and − 0.999645374156623 respectively, which shows that protein becomes unstable when substitution of Glycine takes place at 614 position in replace of Aspartic acid in Table S2 (Supplementary material tables).

#### DDGun

DDGun predicts changes in protein stability caused by mutations using evolutionary traits. A positive G value indicates a potential benefit, whereas negative G values indicate that this substitution is potentially harmful. The CFTR D614G G value of -0.2 suggests that there may be less stability because of the mutation in Table S2 (Supplementary material tables).

#### DUET

DUET uses the change in folding free energy (G) that comes from a protein mutation. A destabilizing mutation, according to DUET, may cause functional changes in the protein that may be significant for diseases. The mutation in CFTR is anticipated to have destabilizing consequences, as calculated by the DUET projected stability change (G), at a rate of 0.8966 kcal/mol (Supplementary Fig. 3) and Table S2 (Supplementary material tables).

#### SDM

A computer method called SDM (Site-Directed Mutator) was used to forecast how mutations affect the stability of proteins. It assesses the effects of single-point mutations on the stability of a protein sequence. The protein is said to be destabilized upon mutation, according to the SDM projected stability change for CFTR D614G, which was − 0.1 kcal/mol in Table S2 (Supplementary material tables).

#### MAESTRO web

MAESTRO is an online program that predicts alterations in protein stability caused by mutations using a multi-agent machine learning system. It was determined that the specific mutation would impact the stability of the protein. The MAESTRO site forecasted a destabilizing mutation, as indicated by the − 0.004 kcal/mol change in overall stability (G value). Furthermore, it was found that the cpred, or confidence estimation, was 0.860, indicating high reliability in Table S2 (Supplementary material tables).

#### DynaMut

DynaMut is a computational tool that forecasts the effects of mutations on proteins and offers information on their dynamics and stability. It analyzes the protein molecule dynamics using normal mode techniques to determine how mutations affect protein function and disease risk. Following a mutation, the expected change in Gibbs free energy was − 1.326 kcal/mol, suggesting a decrease in the stability of the CFTR protein. According to the modified protein's G ENCoM (Elastic Network Contact Model) value of − 0.147 kcal/mol, it is destabilizing. The CFTR protein may have become more flexible after mutation because of the smaller size of the amino acid substitutions. The difference in vibrational entropy energy between the wild-type and mutant forms of the CFTR protein, calculated as SVib ENCoM, was 0.184 kcal.mol-1 K-1 (Supplementary Fig. 4, 5, 6, 7,8) and Table S2 (Supplementary material tables). Calculations over the first 10 non-trivial modes of the molecule were used to calculate the atomic fluctuations, which yielded the amplitude of the absolute atomic motion. Visual representations of the atomic fluctuations are shown in Table S2 (Supplementary material tables). The atomic fluctuations of the first 10 important molecular modes of the CFTR protein were calculated. The deformation energy over the first 10 nontrivial molecular modes was calculated to determine the local flexibility of the CFTR protein.

### Prediction of the structural and functional effect of single nucleotide substitutions

MutPred2 is an online tool, which is used to predict whether an amino acid substitution is pathogenic or benign. MutPred2 scored our variant D614G as 0.855, which shows this variant results in loss of helix and altered metal binding of the protein. This was ultimately altering the transmembrane protein.

### Prediction of physiochemical properties of protein

The mutated as well as nonmutated CFTR proteins’ physiochemical properties were assayed using ProtParam tool, which provides valuable structural and functional properties of the protein such as molecular weight, instability index, theoretical pI, aliphatic index, and GRAVY. By adding the average isotopic masses of the given amino acids, ProtParam determines the molecular weight of the protein. The CFTR protein is projected to have a molecular weight of 168,141.57 for the normal protein and 168,083.54 for the mutant protein, which indicates that it is a rather large protein with both a complicated structure and significant biological functions. The instability index below 40 is typically regarded as a reliable indicator of protein stability. Both proteins' instability indices show that they are only moderately stable, with little variation in mutation upon projected stability. Due to the presence of aliphatic amino acids, the protein in question is likely to be thermostable, as shown by the Aliphatic Index value of 102.82 produced by ProtParam. The protein possesses a net positive charge at pH values below 8.91 and a net negative charge at pH values above 8.91, according to the theoretical pI value of 8.91 for non-mutated CFTR. The CFTR D614G variation would behave similarly, being positive at pH levels below 8.94 and negative at higher pH. This knowledge is essential for comprehending how the protein behaves in diverse biological settings. The GRAVY, which in our case is obtained as 0.024 and 0.026 for standard and mutant CFTR, respectively, shows the hydropathicity of protein structure. It implies that the protein is not strongly hydrophobic but does have a small affinity for hydrophobic interactions. All this information is crucial in predicting the functionality of protein in biological functions in Table S3 (Supplementary material tables).

### Predicting the impact of single nucleotide substitution on PTM sites

MUsite Deep server was used to predict the posttranslational modification in the protein. At the 614 positions, there is no post-translation modification available.

### Predicting the effect of deleterious single nucleotide substitutions on protein properties

The amino acid aspartic acid shifts to glycine at position 614, according to the HOPE server. The schematic depiction of the original (left) and mutant (right) amino acid is displayed in (Supplementary Fig. 9). The side chain, as seen in black, is different, but the backbone is the same. In comparison to the wild residue, the mutant residue is more hydrophobic and smaller. The remnant of the mutant. The NBD1 domain contains the altered residue, which is crucial for the binding of other molecules. The mutation may have an impact on the protein's ability to bind to other molecules, which could have an impact on the protein's functionality.

### Analysis of structure validity of CFTR

The modeled CFTR proteins were subjected to structure validation using Ramachandran plots ERRAT and VERIFY 3D. The comparative outcomes of these validations are presented in Table S4 (Supplementary material tables). The majority of the protein's backbone torsion angles appear to be within optimum ranges given that more than 80% of the amino acid residues in both protein structures are located in this region. This is a potent sign of a protein structure of excellent quality (Supplementary Fig. 10) and in Table S4 (Supplementary material tables). Based on non-bonded atomic interactions, the ERRAT score, which is presented as a percentage, indicates the overall quality of the protein structure. Both CFTR mutant and wild-type protein structures have good levels of overall quality, according to the ERRAT score, which shows that most non-bonded atomic interactions fall within the predicted range. The majority of the CFTR mutant proteins can be seen below the substantial error levels in the residue-by-residue predicted graphs shown in Table S5 (Supplementary material tables) and (Supplementary Fig. 11). By examining whether the 3D model and the protein's own amino acid, sequence agree, Verify3D is a technique for evaluating the quality of protein structures. It shows how closely the model fits the experimental data. The relevant amino acid sequences could not be adequately matched by either of these protein structures, since less than 80% of the amino acids scored >  = 0.1 in the 3D/1D profile (Supplementary Figs. 12, 13).

### Secondary structure validation

Using GOR IV, the secondary structure of both proteins was predicted. It was discovered that both proteins included 37.64 percent random coils, 18.18 percent extended strands, and 44.19 percent alpha helix. The protein sequence's projected secondary structure was unaffected by a single amino acid change. Figure 5 shows a thorough graphic representation of the secondary structure (Supplementary Fig. 14).

### Modelling of variants of CFTR protein

#### TM-align

The average distance between matching atoms in two proteins’ aligned structures is measured by RMSD. The RMSD value in this alignment is 2.23, indicating that the average distance between atoms in the structures of the wild-type and mutant proteins is 2.23 angstroms. A lower RMSD value indicates that the proteins are more structurally similar or more comparable in their three-dimensional structure. The TM-score, which ranges from zero to one, is a numerical indicator of how structurally similar two proteins are, with higher values denoting greater similarity. The TM-score in this alignment is 0.88043. With 1.0 being a perfect match, the high TM score indicates that the wild-type and mutant proteins share plenty of structural similarities (Supplementary Fig. 14).

#### PyMOL

**Fig. 1:**
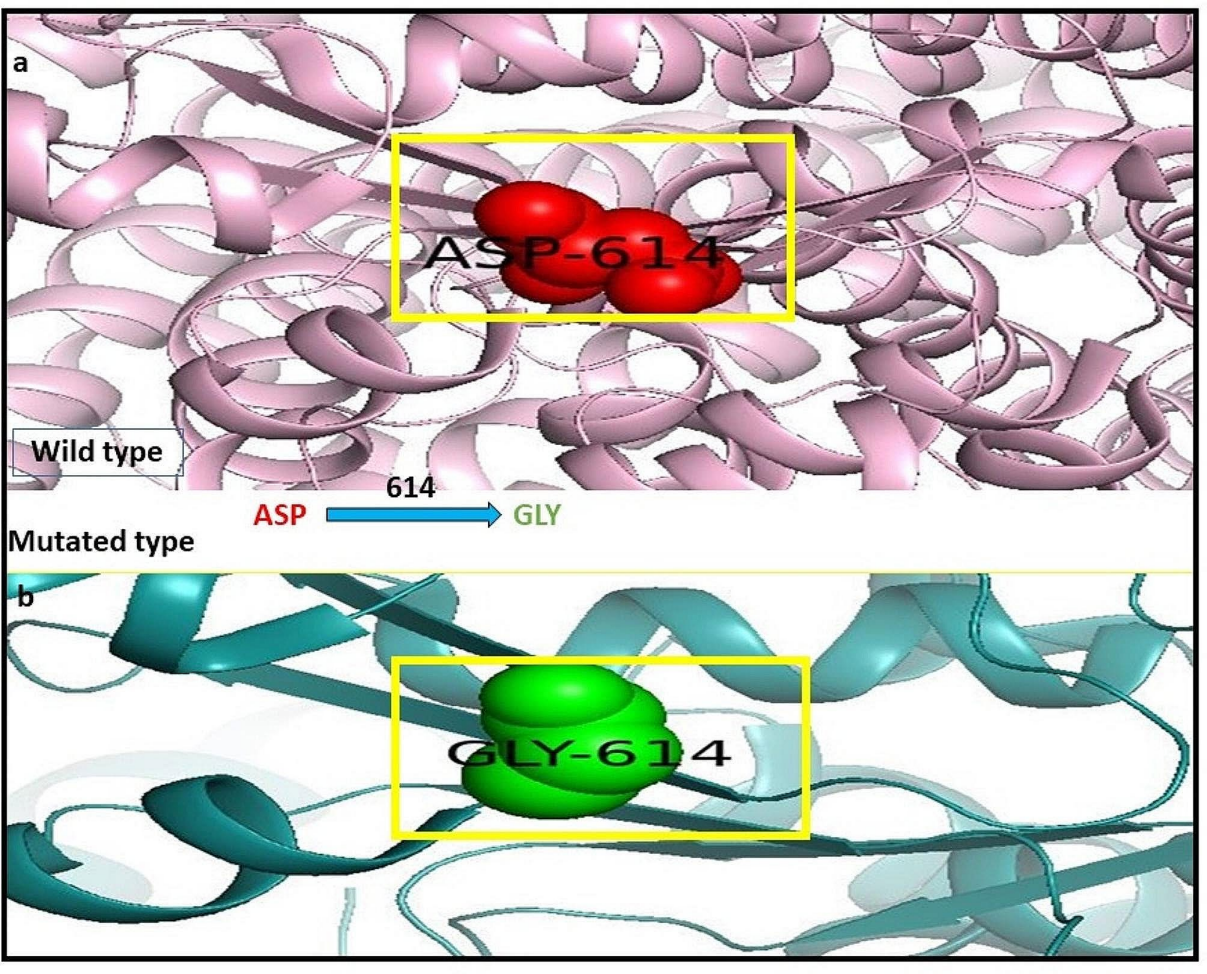
**a**–**b**: CFTR protein structure: **a** Wild type CFTR represents green area. **b** Mutated CFTR (D614G) is represented in the yellow area

PyMOL tool is used for the visualization of the 3D structure of the wild and mutated type of CFTR protein. Below are the figures showing that in wild-type CFTR protein at 614 position aspartic acid amino acid shown in red is changed into glycine amino acid shown in yellow color in Fig. 1.

### Molecular docking of multiple ligands with mutated *CFTR* variant (D614G)

**Fig. 2:**
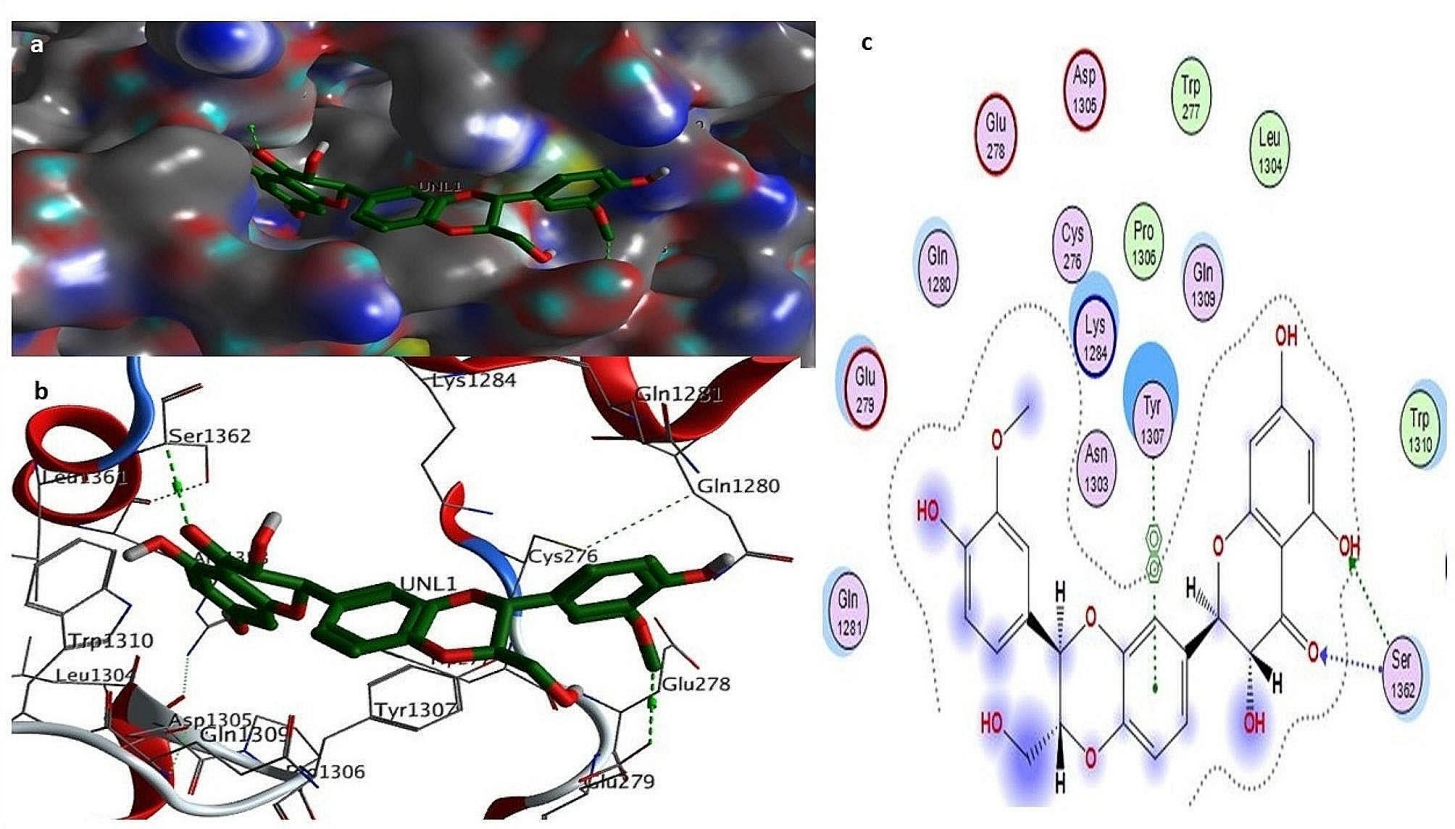
**a**–**c**: Docking complex of mutated *CFTR* (D614G) structure with demothoxycurcumin: **a** Docking complex of the protein–ligand surface of human mutated CFTR (red surface) with (dark purple color). **b** Indicate interacting residue of mutated CFTR structure with ligand. Red and grey balls show the active binding site of proteins. **c** The 2D diagram represents protein–ligand interacting residues indicating hydrogen bonding

**Fig. 3:**
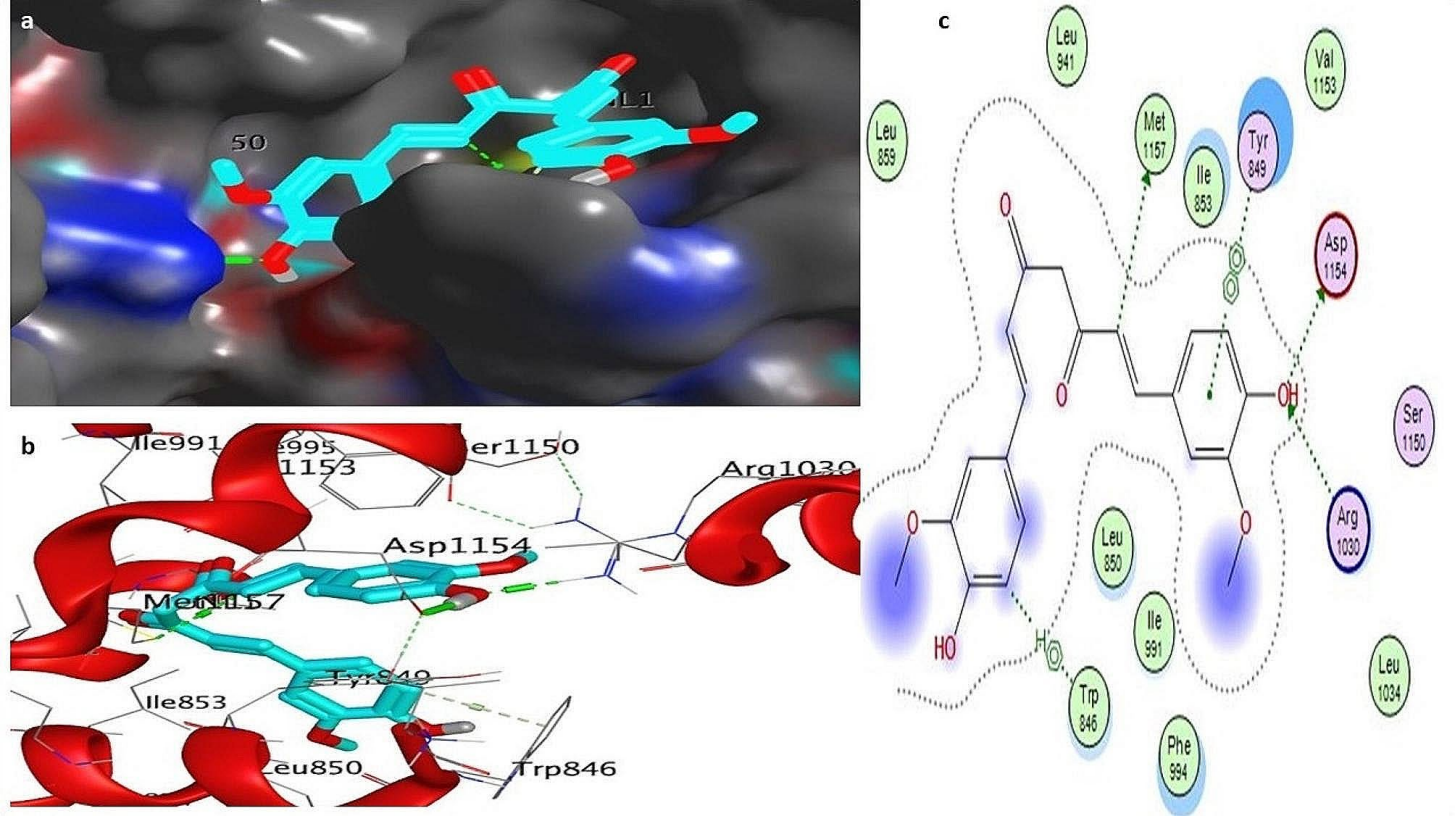
**a**–**c**: Docking complex of mutated CFTR (D614G) structure with silibinin: **a** Docking complex of the protein–ligand surface of human mutated CFTR (red surface) with silibinins (dark green color). **b** Indicate interacting residue of mutated CFTR structure with ligand. Red and grey balls show the active binding site of proteins. **c** The 2D diagram represents protein–ligand interacting residues indicating hydrogen bonding

**Fig. 4:**
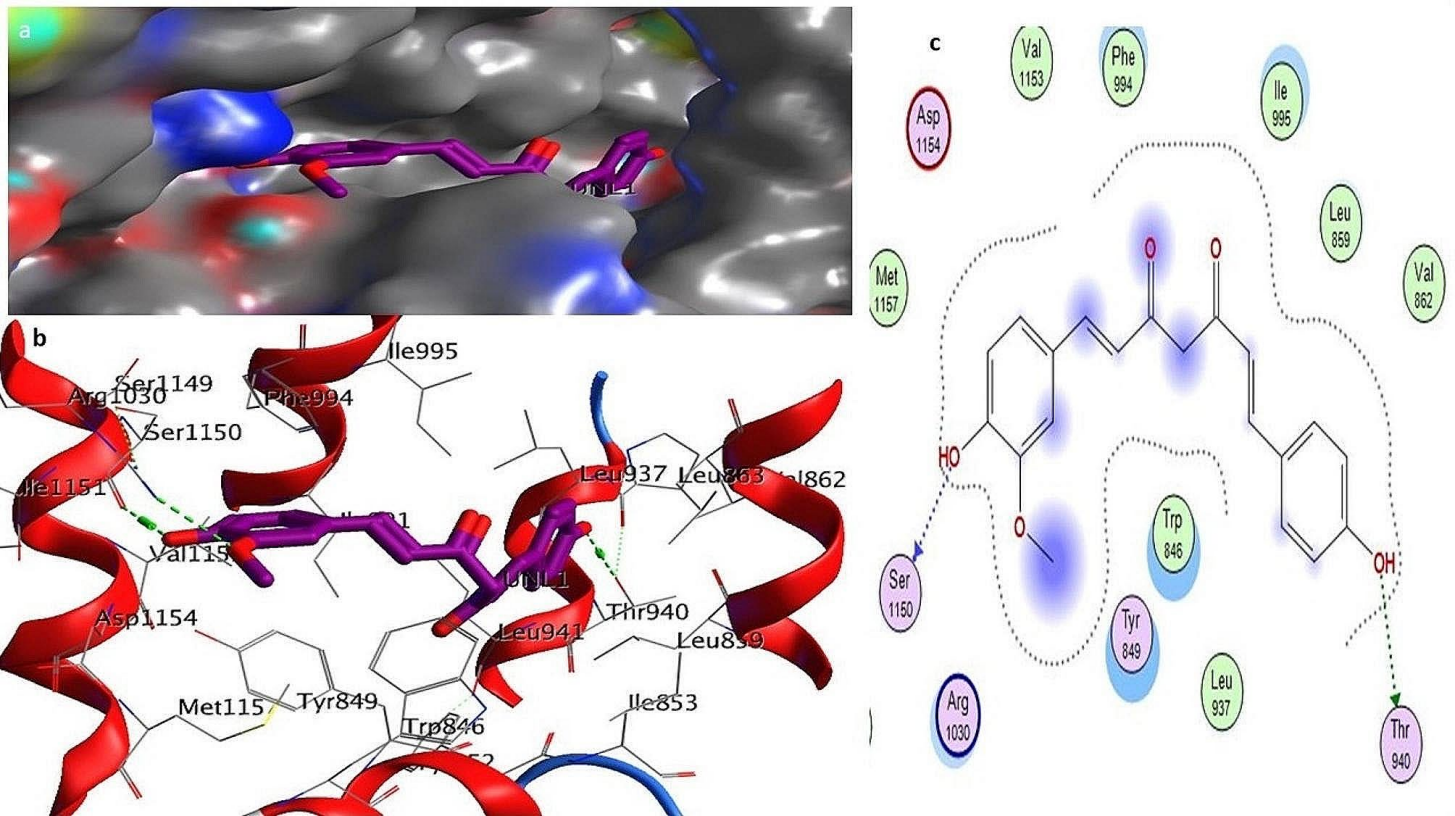
**a**–**c**: Docking complex of mutated CFTR (D614G) structure with curcumin: **a** Docking complex of the protein–ligand surface of human mutated CFTR (red surface) with curcumin (purple bolls). **b** Indicate interacting residue of mutated CFTR structure with ligand. Red and grey balls show active binding sites of proteins. **c** The 2D diagram represents protein–ligand interacting residues that indicate hydrogen bonding

**Fig. 5:**
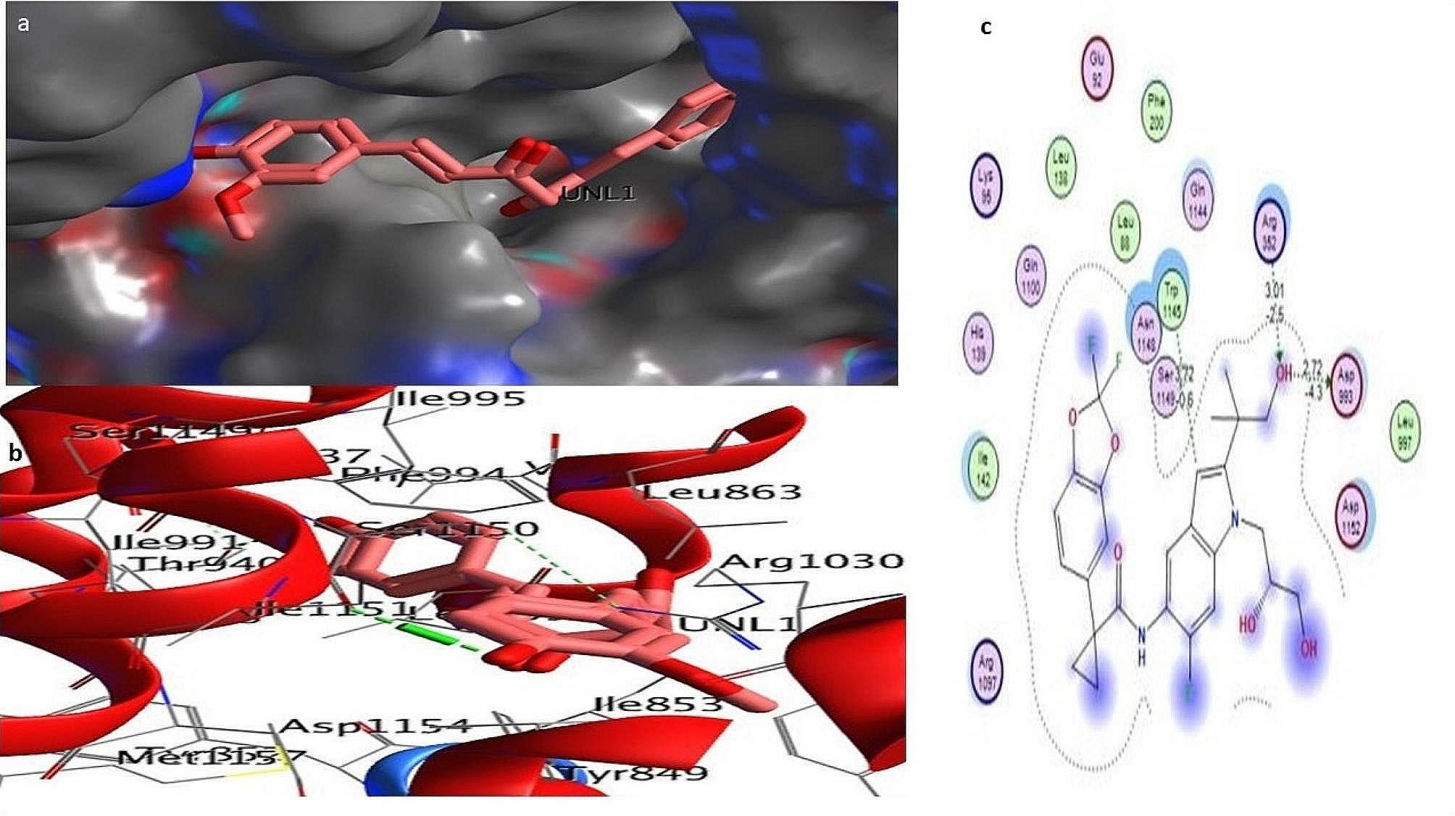
**a**–**c**: Docking complex of mutated CFTR (D614G) structure with Trikafta: **a** Docking complex of protein–ligand surface of human mutated CFTR (red surface) with Trikafta (cyan, green color). **b** Indicate interacting residue of mutated CFTR structure with the reference drug Trikafta. Red and grey balls show active binding sites of proteins. **c** The 2D diagram represents protein drug interacting residues indicating hydrogen bonding

To investigate the influence of a particular mutation on the structure and function of the CFTR protein, molecular docking was performed with three different natural compounds. This was done by calculating the binding energy of the ligands with the mutated structure of the CFTR protein. Below are the different ligands and their interaction with the mutated D614G variant. Auto-DOCK scores for these ligands against mutant human *CFTR* (D614G) are − 6.20 kcal/mol for Silibinins, − 6.5 kcal/mol for Curcumin, − 5.85 kcal/mol for Demethoxycurcumin, − 5.50 kcal/mol for the reference drug Trikafta. Among all the natural compounds we used silibinins compound shows the maximum binding affinity with the mutated D614G variant of CFTR. The docking results showed that the docking score of silibinin, curcumin, and demethoxycucumin were the highest among the natural ligands used in this study. The interaction of the silibinin in the active site of the mutated model protein showed that the silibinin was well established and had a significant and maximum interaction with the key amino acids of the protein. Investigation of the interaction of the silibinin with the CFTR showed that the carbonyl and hydroxyl group of silibinin could form hydrogen bond with the amino acid’s residues; Glu193, Arg 1097. The interaction of the Demethoxycurcumin and curcumin in the active site of the mutated model protein showed that the Demethoxycurcumin and curcumin were also having good interaction with the key amino acids of the protein. Investigation of the interaction of these two ligands Demethoxycurcumin and curcumin with the CFTR showed that the carbonyl and hydroxyl group of these ligands could form two and one hydrogen bond with the amino acid’s residues: Lys 1041, Arg 1078, and Glu 193 respectively, see Figs. 2, 3, 4, 5a, c and Table S6 (Supplementary material tables).

The three of them fail to meet the criteria of Lipinski's rule of five with regard to the specified parameters. For each ligand, the permissible ranges for molecular weight < 500, the number of hydrogen bond acceptors and donor are less than 5, and 10, respectively. Molecules with a TPSA of 140 Å2 or greater would exhibit low fractional absorption (< 10%), while those with a TPSA of 60 Å2 or less would demonstrate high absorption (> 90%). The topological Polar Surface Area (TPSA) and %age of the natural ligands showed the preference as demethoxycurcumin > curcumin > silibinin. A medication candidate with a lipophilicity value between 0 and 5 would be suitable. Demethoxycurcumin (3.0), silibinin (1.59), and curcumin (1.23), according to the lipophilicity (LogPo/w) study, are significantly more lipophilic than the other natural compounds. The projected results indicate that oral and intestinal absorptions are achievable, although more clinical research is needed. Understanding that a medicine is a substrate for CYP and P-gp is also important for its proper bodily metabolism. Together, the two tiny chemicals strengthen the body’s defences against organ and tissue damage. Almost 50 to 90 percent of medicinal compounds are either one of the five CYP substrates. CYP3A4 is the most significant of the five isoforms. A substance will have a hazardous effect on the body if it inhibits any of these enzymes. Whereas demethoxycurcumin and silibinin inhibit CYP3A4, curcumin does not inhibit it in Tables S7,S8 (Supplementary material tables).

### Analysis of molecular dynamic simulation of mutated CFTR protein with different natural compounds

We were verified the ligand binding modalities and the stability of the protein–ligand complexes using MD simulations and the Desmond program. The 200 ns simulations were performed using the top dock scorer, which included the CFTR (D614G) complexes together with three natural ligands including silibinin, curcumin and demethoxycurcumin. The RMSD figure showed the root mean standard deviation of the protein–ligand complex’s C-α backbone during the simulation.

**Fig. 6:**
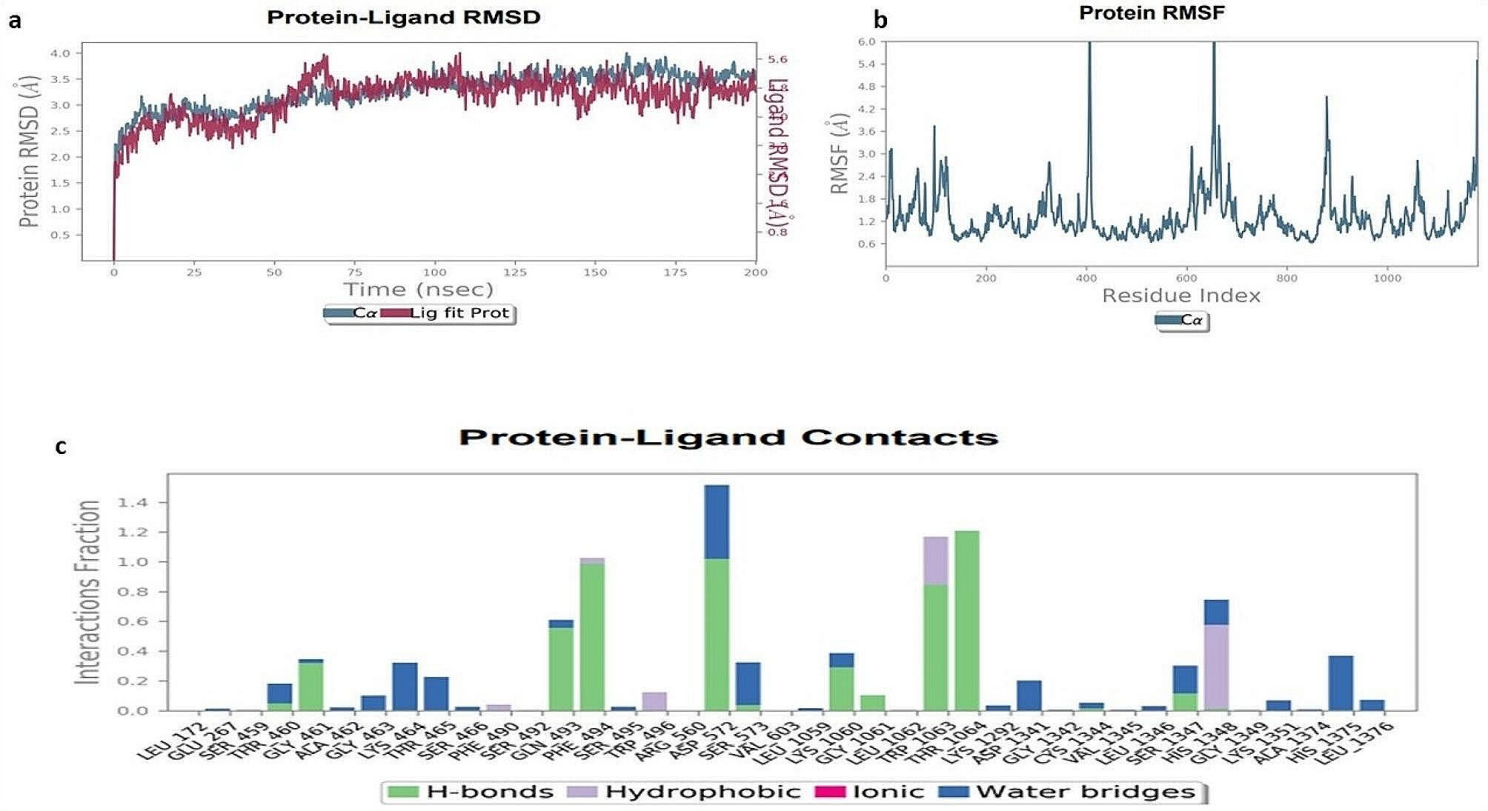
**a**–**c**. MD simulation analysis of the Silibinin with CFTR (D614G) complex. **a** RMSD protein ligand graph. **b** CFTR protein RMSFs. **c** The protein–ligand contact histogram

Using MD for 200 ns, the C-α backbone of the CFTR protein in the Silibinin complex was simulated. The ligand was depicted in pink, and the C-α backbone's RMSD within the light blue range. The protein–ligand complex was found to be stable throughout the simulation, with observations made up to 200 ns. The simulation demonstrated the persistence of the CFTR protein, as seen by its C-α backbone of 3.5 Å, which observed minor fluctuation between 2.5 nm and 3.6 nm after 60 ns and then remain constant. The simulation results' RMSF showed that the residues of ARG-550 and GLU-400 varied above 6.0 Å. During the simulation, there was no ligand association seen with these protein residues. In the Silibinin-CFTR complex, the crucial residue of THR-1064 was shown to be directly involved in forming a hydrogen bond with the OH-benzne ring. The protein's crystal structure demonstrates how the Silibinin-CFTR complex interacts with residues including ASP-572, GLN-573, SER-1375, and LYS-1060 to generate stable hydrogen bonds through water-mediated interactions. Furthermore, Silibinin engages in hydrophobic bonding with water-mediated contacts with TRP-1063 and PHE-494. During the simulation, additional interactions were seen with different amino acids. Nonetheless, throughout the simulation, the contact with the receptor's GLU-267, THR-460, and ALA-462 residues remained constant and strong as shown in Fig. 6a–c.

**Fig. 7:**
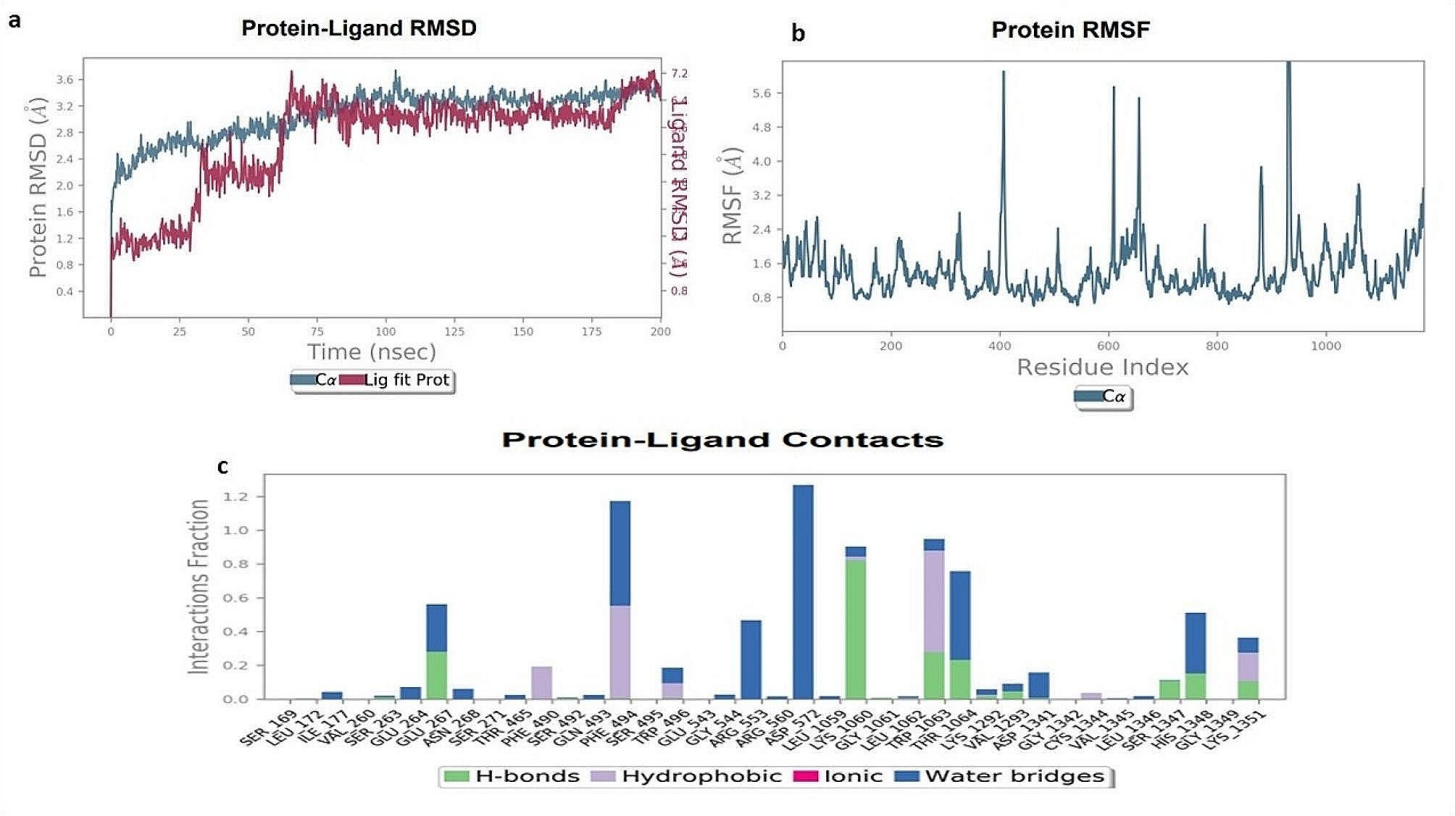
**a**–**c**. MD simulation analysis of the Curcumin with CFTR (D614G) complex. **a** RMSD protein ligand graph. **b** CFTR protein RMSFs. **c** The protein–ligand contact histogram

The CFTR-Curcumin complex was shown to be stable throughout the simulation, with observations up to 200 ns, according to the RMSD plot. With a C-α backbone of 3.6 Å, the CFTR protein was shown to have remained stable over the simulation, which observed minor fluctuation between 1.2 nm and 3.5 nm after 60 ns and then remain constant. The simulation findings' RMSF showed that GLU-400 and GLY-600 residues fluctuated above 5.6 Å. Throughout the simulation, the CFTR-curcumin complex interacts with residues like ARG-1437 through water-mediated interactions, forming stable hydrogen bonds. Nonetheless, throughout the simulation, the contact with the receptor's residues remained constant and strong as shown in Fig. 7a–c.

The RMSD figure indicated that the CFTR-Desmethoxycurcumin combination was not found to be stable during the simulation, with observations up to 200 ns. With a C-α backbone of 3.6 Å, the CFTR protein was shown to have remained stable over the simulation, which observed minor fluctuation between 2.4 nm and 3.6 nm after 125 ns and then remain constant. The ligands of Desmethoxycurcumin were observed major fluctuations in the complex structure and did not shown stability within protein CFTR mutated structure. The simulation results showed that the residues of GLU-400, GLY-600, and GLY-801 fluctuated over 5.6 Å, as shown by the RMSF. During the simulation, it was observed that these protein residues were not connected to a ligand. When the CFTR-Curcumin complex interacts with residues like GLU-267, THR-1064, and HIS-1348 through water-mediated interactions, stable hydrogen bonds are formed. Moreover, during the simulation, Curcumin interacts with TRP-496 and PHE-494 through hydrophobic bonding and water-mediated interactions; (see Fig. 9). But throughout the simulation, the contact with the receptor's ILE-177, SER-263, GLU-264, and ASN-268 residues remained constant and strong. The best MD simulation results indicated Silibinin and Curcumin with CFTR protein indicate both complex stable throughout the simulation and no significant fluctuations in (supplementary Fig. 15a–c).

### Analysis of radius of gyration (rGyr) of mutated CFTR protein with different natural compounds

**Fig. 8:**
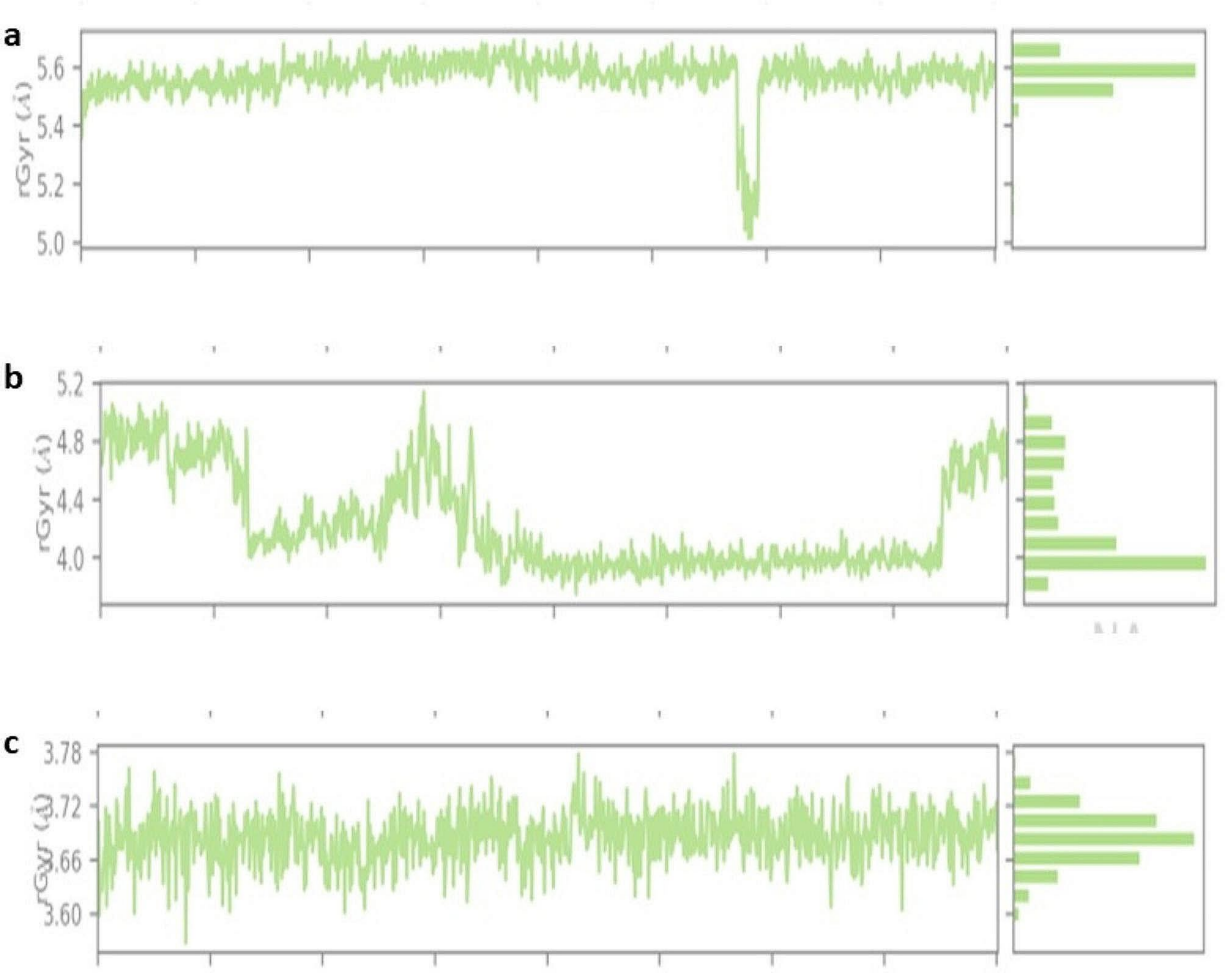
**a**–**c**. Gyration radii of the complex. **a**. Silibinin ligand with CFTR protein gyration radii complex graph. **b** Curcumin ligand with CFTR protein gyration radii complex graph. **c** Demethoxycurcumin with CFTR protein gyration radii complex graph

**Fig. 9:**
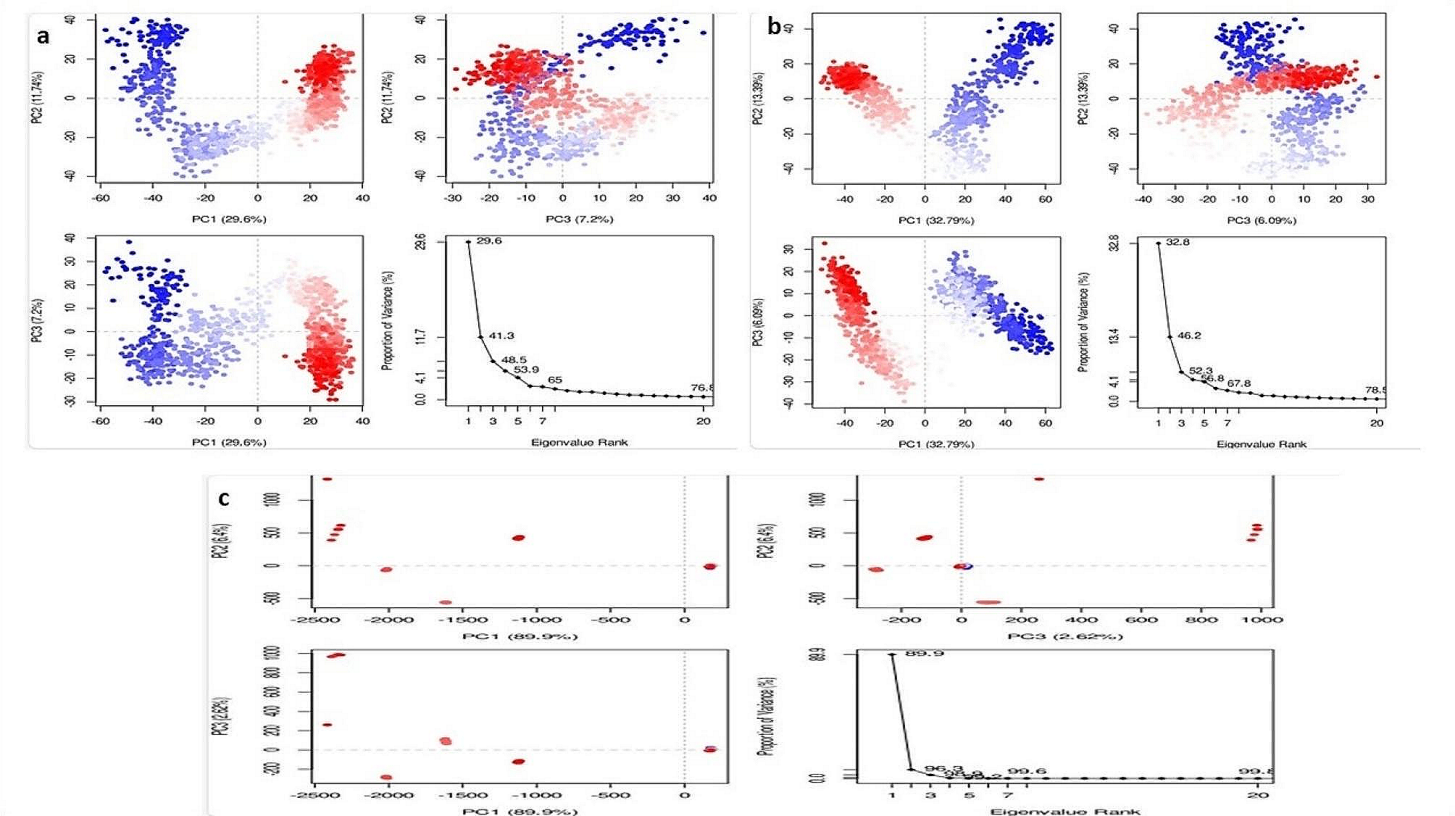
**a**–**c**. Principle Component Analysis. **a** PCA of Silibinin with CFTR (D614G) complex. **b** PCA of Curcumin with CFTR (D614G) complex **c** PCA of Demethoxycurcumin with CFTR (D614G) complex

The whole molecular dynamics (MD) simulation trajectory set was used to compute the rGyr parameter, which was used to assess the stability of the Silibinins—CFTR (D614G) complex. When the protein maintains its stability throughout the simulation, it has a stable rGyr value of 5.4 Å. During the 200 ns simulation, the structural compactness, or folding changes of silibinins with protein complexes were demonstrated using the RGyr parameter. Interestingly, no discernible variations were found for the complicated see Fig. 9a. The stability of the Demethoxycurcumin-CFTR (D614G) complex was evaluated by using the rGyr parameter, which was obtained from the complete molecular dynamics (MD) simulation trajectory set across the 200 ns simulation. The protein has a steady rGyr value of 3.72 Å when it was not stable throughout the simulation. Figure 9(b) for the complex see showed no discernible differences. Based on a simulation lasting 200 ns, the stability of the Curcumin—CFTR (D614G) complex was assessed using the rGyr parameter. A consistent rGyr value of 4.8 Å suggests that the protein maintained its stability following some modest fluctuations during the experiment. Interestingly, there were no discernible variations for the complex. These findings imply that following binding, protein–ligand complexes stay stable, as seen in Fig. 8c.

### Calculation of MM-GBSA Binding free energy calculation

After molecular simulation, the ΔGbind values were determined using the MM-GBSA method and shows the degree of binding strength between the protein and ligand complexes. The complexes generated by CFTR with Silibinins, Demethoxycurcumin, and Curcumin had average ΔGbind values of − 124.55, − 62.637497, and -39.111, respectively. As can be seen in Table S9 (Supplementary material tables), a significantly negative number indicates a larger binding affinity between the two.

### Principle component analysis of mutated CFTR protein with compounds

Silibinins, and Curcumin were the most stable ligands among the selected compounds during the MDS process, according to PCA data. The percentage of variance for each component is displayed in three separate parts of PCA eigenvalue plots. For instance, different regions of the silibinins-CFTR protein in variants in PC1, PC2, and PC3 contribute, respectively, 29.6%, 11.74%, and 7.2% of the total variance. A graphic representation of these data may be found in Fig. 9. The PCA results for Curcumin; 32.9%, 13.39%, and 6.09%. The PCA result indicating silibinins and curcumin with the CFTR protein had the highest score in Fig. 9a–c.

## Discussion

A protein's three-dimensional shape determines how it functions within the cell, and how any alteration in the sequence of amino acids might cause illness. The protein that oversees the movement of chloride ions across the cell membrane; is made according to instructions provided by the *CFTR* gene (Vergani et al. 2003). Cystic fibrosis results from abnormalities in the CFTR protein. Bioinformatics tools assist in predicting the structural and functional effects of mutation on CFTR by utilizing a variety of techniques and algorithms. Because a protein's tertiary structure is essential for its functional complexity, alterations in its amino acid sequence can cause structural changes and, as a result, lead to the development of disease (Khedkar et al. 2007).

The primary focus of this study was to identify and determine the harmful effects of these single nucleotide substitution (D614G) mutations, shedding light on their potential impact on the overall functionality of the CFTR protein. Furthermore, docking of the protein with natural ligands helps us determine which ligands show the best binding affinity with CFTR (Brindha et al. 2020). For this study, we obtained our information from the CFTR-2 annotation databases, which comprise almost 779 missense variants. We also used ClinVar to provide a reliable interpretation of our variant. For this study, we chose the D614G missense variant because of its pathogenicity in which aspartic acid amino acid at position 614 changes to glycine amino acid. We used a range of different computational tools to understand the structural and functional alterations that the single nucleotide substitutions cause in the protein CFTR. First, the protein CFTR was subjected to functional and structural study analysis using well-known tools like SIFT, PROVEAN, PolyPhen-2, and PANTHER-PSEP. SIFT, for instance, discovered that the D614G mutation might be harmful to the body. PROVEAN and PolyPhen-2 demonstrated the deleterious effects of the substitution of glycine for aspartic acid at position 614 (supplementary Figs. 2, 3, 4, 5, 6, 7, 8). It changes the protein’s structural makeup. Moving towards structural stability, the CFTR protein's stability modifications were predicted using I Mutant 2.0 and the MUpro server. I-Mutant 2 shows that the CFTR protein's stability was lowered by the D614G mutation. MUpro demonstrates that the stability of protein was reduced by this variation. The following servers were used to compare and analyze the structural stability of protein: MutPred, MusiteDeep, Pfam, and Project HOPE. MutPred plays a critical role in analyzing the impact of single nucleotide changes on the structure and function of the CFTR protein when researching the subject. Furthermore, our prediction results were very consistent with earlier investigations (Ghosh et al. 2021). Five functional domains are present in the CFTR, according to the Pfam database. The CFTR protein's modest alterations between the wild and mutant forms are revealed by secondary sequencing analysis using various in silico tools. Protein structure and interaction can be impacted by mutations that effects the hydrophobicity, charge, and size of amino acid residues (supplementary files 9, 10, 11, 12, 13, 14).

Using the PyMOL software, was performed to create models for mutant variants of the CFTR protein in Fig. 1a–b. The mutant model has undergone subsequent molecular docking analysis using Discovery studio software to evaluate the binding affinity of mutant CFTR with the natural ligands (Sakhawat et al. 2023). Our study investigated the effectiveness of different natural compounds along with the approved drug Trikafta, specifically targeting patients with the D614G CFTR variant. We sought to identify the most promising combination of drugs with mutated proteins for optimal results. We selected silbinins, demethoxycurcumin, and curcumin as they have good synergetic effects with the approved drug Trikafta against the D614G variant. While pharmaceutical modulators such as Trikafta work at many levels (correcting and potentiating CFTR function), natural substances frequently have a single mechanism (either corrector or potentiator). This contrast emphasizes the importance of combination techniques when evaluating natural chemicals as viable therapeutics (Li et al. 2024). Natural chemicals are typically thought to have fewer adverse effects, however complete safety profiles are missing. Pharmaceutical modulators, on the other hand, might have serious negative effects, notwithstanding their effectiveness. Among all the ligands silibinin with 6.20 kcal/mol shows the maximum binding affinity with the mutated structure of CFTR in Fig. 2, 3, 4, 5a–c. Table S6 (Supplementary material tables) displays the outcomes of the bioavailability screening for the medication R*- and multiple ligands. All natural compounds follow Lipinski's rule states that substances that have less than 10 hydrogen bond acceptors (HBAs) and a molecular weight of less than 500 are usually well-absorbed by the body. However, Lipinski's requirements were not met by the reference medicine medication R* due to higher molecular weight. The study of a drug's absorption, distribution, metabolism, and excretion from a biological system is known as pharmacokinetics. The proportion of a medication's therapeutic impact that enters the bloodstream is known as oral bioavailability. The bioavailability scores of the various ligands vary from 0.55 to 0.56, indicating good oral bioavailability, following Lipinski’s rule of five. Furthermore, our prediction results were very consistent with earlier investigations (Iazzi et al. 2023). In Table S7 (Supplementary material tables) shows, that all ligands, as well as the R*-drug, were found to have gastrointestinal absorption potential. Curcumin, Silibinin as well as the reference drug R*, are unable to enter the BBB and this may have positive effects on the central nervous system. Only drugs targeting the CNS are required to penetrate the BBB. Through efflux activity, membrane transporters known as P-glycoproteins (P-gps) it limit the cellular uptake of their substrates from blood circulation into the brain. Since all ligands were shown to be non-substrates of P-gps, P-gp efflux activity may not have an impact on them, leading to an increase in serum concentration and leads to beneficial therapeutic effect. In contrast, the reference drug R*, which is a substrate of the protein P-gp, may have significantly lower serum concentrations, leading to therapeutic failure if used alone, unless combined with P-gp inhibitors. Furthermore, our prediction results were very consistent with earlier investigations (Heo et al. 2022; Hassan et al. 2023; Ullah et al. 2023a, b; Ullah et al. 2023a, b).

Table S8 (Supplementary material tables) shows the effect of cytochrome P450 (CYP450) induction or inhibition on drug bioavailability. CYP450 enzymes metabolize clinically relevant drug interactions. Inhibitors either prohibit CYP450 enzymes from functioning or limit the pace of enzyme-catalyzed processes. Furthermore, our prediction results were very consistent with earlier investigations (Iacopetta et al. 2023). As a result, medication metabolism in the body is reduced, and the risk of toxicity increases. The metabolism of natural compounds was tested against CYP3A4 isoforms. Trikafa reference drug R* either inhibits these CYP3A4 enzymes. For example, R* may inhibit CYP3A4 to induce toxicity if it suppresses CYP3A4 expression. Trikafa and other ligands’ pharmacokinetics, including how they are absorbed, distributed, metabolized, and removed, can be better understood. With MD simulations for virtual screening, diseases resulting from protein misfolding can be identified by analyzing the stability and ligand binding kinetics of protein–ligand complexes (Odera et al. 2018; PERVAIZ et al. 2021; Nawaz et al. 2024). The system was entirely stabilized with an RMSD of 3 Å, according to the RMSD graph of Silibinin-CFTR (D614G). Curcumin-CFTR (D614G) showed modest oscillations with an RMSD of 3.6 Å, with changes observed between 50 and 75 ns. Apart from Demethoxycurcumin, the protein–ligand RMSD did not change over the last 200 ns of the experiment, when equilibrium was reached. The amino acids displayed RMSF results in both protein–ligand complexes, indicating a stable protein structure. The ligand in the protein–ligand interaction shows a strong relation with several amino acids and does not deviate greatly from its original form. Our work investigated the interactions between the altered CFTR structure and three natural substances using an MD simulation trajectory. The MD simulation, also revealed silibinins and curcumin ligands see Fig. 6, 7a–c, indicating that the structure of the altered CFTR-ligand bound complex was quite stable than Demethoxycurcumin (supplementary Fig. 15). Based on a simulation lasting 200 ns, the stability of the silibinins and curcumin—CFTR (D614G) complex was assessed using the rGyr parameter. A consistent rGyr value of 3.8 Å suggests that the protein maintained its stability following some modest fluctuations during the experiment. Interestingly, there were no discernible variations for the complex. These findings imply that following binding, protein–ligand complexes stay stable, as seen in Fig. 8a–c. To evaluate the results of MM-GBSA, one must understand the computed energy components and how they affect the overall free energy change in a molecular system. This is often the case when studying the interaction between proteins and ligands. The larger negative binding energy of the ligands of silibinin and curcumin indicates that binding is thermodynamically advantageous in Table S9 (Supplementary material tables). The PCA result indicating silibinins and curcumin with the CFTR protein had the highest score in Fig. 9a–c. For better accuracy, it is advisable to use multiple tools and make informed decisions by comparing the results. Combining computational tools with the wet lab study provides a better understanding of the mutation in the CFTR protein. Furthermore, our prediction results were very consistent with earlier investigations (Zeng et al. 2016). This work highlights the potential of silibinins and curcumin natural derivatives as therapeutic medicines for cystic fibrosis (CF) associated with mutant CFTR proteins by thorough molecular docking, molecular dynamics simulations, and ADME studies. Strong binding affinities were shown by these natural compounds for the mutated CFTR protein, resulting in crucial interactions as well as advantageous pharmacokinetic and toxicological characteristics.

In conclusion, in silico studies demonstrate the potential of silibinins and curcumin as innovative therapeutic agents for cystic fibrosis, particularly for the D614G mutation. Their ability to increase CFTR function while reducing cellular stress and inflammation, together with their favorable safety profile and accessibility, makes them valuable additions to cystic fibrosis treatment options. Further experimental and clinical validation will be required to fully realize their potential and include them into effective therapy regimens.

### Supplementary material

Supplementary Material 1.

Supplementary Material 2.

## Data Availability

The dataset analyzed during the current study is easily available in the NCBI-database (CFTR protein) (www.ncbi.nlm.nih.gov) and PubChem database for ligands. 1. Identification of Protein (CFTR protein: Accession No# P13569.3), Identification Natural compounds, 2. (Silibinin: Accession No# 31553), 3. (Curcumin: Accession No# 5280961), 4. (Demethoxycurcumin: Accession No# 5469424), 5. (R* (Trikafta): Accession No# 165363555). No data associated in the manuscript.
