# Supplementary material for: Silibinins and curcumin as promising ligands against mutant cystic fibrosis transmembrane regulator protein
Source: AMB Express. 2024 Jul 23;14:84. doi: 10.1186/s13568-024-01742-z (PMC11266341; doi:10.1186/s13568-024-01742-z)
Supplement: Supplementary file 1 — Supplementary Material 1. [file 13568_2024_1742_MOESM1_ESM.docx]

**Supplementary Material Tables**

**Table S1. Functional based prediction**

| **Variant (D614G)** | **Score** | **Prediction** |
| --- | --- | --- |
| **SIFT** | 0.05 | Low confidence |
| **Panther** | 0.85 | Probably damaging |
| **PolyPhen 2** | Close to 1 | Probably damaging |

**Table S2. Structure based prediction**

| **Variant (D 614G)** | **Score** | **Prediction** |
| --- | --- | --- |
| **MutPred** | 0.855 | Destabilizing |
| **MUPro** | -1.73 | Destabilizing |
| **DDGun** | -0.22 | Destabilizing |
| **Duet** | -0.8966 kcal/mol | Destabilizing |
| **SDM** | -0.1 kcal/mol | Destabilizing |
| **MasteroWeb** | -0.004 kcal/mol | Destabilizing |
| **DynaMut** | -1.326kcal/mol | Destabilizing |

| **Table S3. Physical and chemical parameters of standard and mutated CFTR proteins.** | | | | | | | | |
| --- | --- | --- | --- | --- | --- | --- | --- | --- |
| Physical and chemical parameters | | **Normal** | | | **Mutated** | | | |
| **Number of amino acids** | | 1480 | | | 1480 | | | |
| **Formula** | | C_7640_H_12106_N_2000_O_2150_S_55_ | | | C_7638_H_12104_N_2000_O_2148_S_55_ | | | |
| **Theoretical pI** | | 8.91 | | | 8.94 | | | |
| **Molecular weight** | | 168141.57 | | | 168083.54 | | | |
| **Instability index** | | 43.80 | | | 43.85 | | | |
| **Aliphatic index** | | 102.82 | | | 102.82 | | | |
| **Total number of positively charged residues** | | (Asp + Glu): 151 | | | (Asp + Glu): 150 | | | |
| **Total number of negatively charged residues** | | (Arg + Lys): 170 | | | (Arg + Lys): 170 | | | |
| **Grand average of hydropathicity (GRAVY)** | | 0.024 | | | 0.026 | | | |
| **Table S4. Structure validation of standard and mutated CFTR protein** | | | | | | | |  |
|  | | CFTR standard | | | CFTR mutated | | |  |
| Ramachandran plot | | 84.0% | | | 89.2% | | |  |
| ERRAT | | 80.9098 | | | 88.8452 | | |  |
| **Table S5. RC plot scores of standard and mutated CFTR protein** | | | | | | | | |
|  | | | | CFTR standard | | | CFTR mutated | |
| Most favored regions | | | | 84.0% | | | 89.2% | |
| Additionally allowed regions | | | | 14.0% | | | 12.0% | |
| Generously allowed regions | | | | 1.5% | | | 1.1% | |
| Disallowed regions | | | | 0.5% | | | 0.7% | |
| Total | | | | 100% | | | 100% | |

**Table S6. Molecular docking of Multiple ligands (MPLs) and (R*) binding affinities against mutated *CFTR* (D614G) structure with their interacting residues.**

| **Ligands** | **S-score** | **H-Bond** | **Distance** | **Interacting residues** | **Names of residues** |
| --- | --- | --- | --- | --- | --- |
| **Silibinins** | -6.20 kcal/mol | 2 | 2.77 Å,  3.25 Å | Direct Interacting residue | Glu-193, Arg-1097 |
| **Demethoxycurcumin** | -5.85 kcal/mol | 1 | 3.07Å, 3.03 Å | Direct Interacting residue | Tyr1092, Gln-353 |
| **Curcumin** | -6.5 kcal/mol | 2 | 2.92, 2.9 | Direct Interacting residue | Arg-1078, Glu-193 |
| **R*** | -6.4 kcal/mol | 3 | 3.94Å,2.3Å,3.5Å | Direct Interacting residue | Arg-352, Asp-993, Trp-1145 |

**Table S7. Analysis of Drug likeness, pharmacokinetics, and toxicity profile of natural compounds and reference drugs**

| **Ligand names** | **Molecular weight and formula** | **Lipophilicity & solubility** | **Lipinski rule** | **Structure** | **Canonical SMILE** |
| --- | --- | --- | --- | --- | --- |
| **Demethoxycurcumin** | 338.4 g/mol  C_20_H_18_O_5_ | 2.56  Soluble | Yes follow.  0 violation | 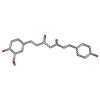 | COC1=C(C=CC(=C1)C=CC(=O)CC(=O)C=CC2=CC=C(C=C2)O)O |
| **Silibinins** | 482.4 g/mol  C_25_H_22_O_10_ | 1.59  Moderately soluble | Yes follow.  0 violation | 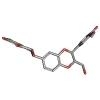 | COC1=C(C=CC(=C1)C2C(OC3=C(O2)C=C(C=C3)C4C(C(=O)C5=C(C=C(C=C5O4)O)O)O)CO)O |
| **Curcumin** | 368.4 g/mol  C_21_H_20_O_6_ | 3.03  Moderately soluble | Yes follow.  0 violation | 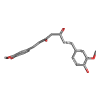 | COC1=C(C=CC(=C1)C=CC(=O)CC(=O)C=CC2=CC(=C(C=C2)O)OC)O |
| **Trikafta ®** | 1433.5 g/mol  C_76_H_82_F_6_N_6_O_15_ | 3.48  Poorly insoluble | Not follow Lipinski rule | 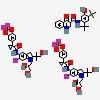 | CC(C)(C)C1=CC(=C(C=C1NC(=O)C2=CNC3=CC=CC=C3C2=O)O)C(C)(C)C.CC(C)(CO)C1=CC2=CC(=C(C=C2N1CC(CO)O)F)NC(=O)C3(CC3)C4=CC5=C(C=C4)OC(O5)(F)F.CC(C)(CO)C1=CC2=CC(=C(C=C2N1CC(CO)O)F)NC(=O)C3(CC3)C4=CC5=C(C=C4)OC(O5)(F)F |

**Table S8. Pharmacokinetics and toxicity profile of natural ligands and Reference drugs**

| **Ligand name** | **PubChem CID** | **Absorption** | **Bioavailability score**  **P-gp substrate** | **CYP3A4 inhibitor** | **Blood brain barrier permeability** |
| --- | --- | --- | --- | --- | --- |
| **Demethoxycurcumin** | 5469424 | High GI absorption | 0.55  No | YES | No |
| **Silibinin** | 31553 | Low GI absorption | 0.55  No | YES | No |
| **Curcumin** | 5280961 | High GI absorption | 0.55  No | YES | No |
| **Trikafta ®** | 165363555 | High GI absorption | 0.55  - | - | - |

**Table S9. MM-GBSA Binding free energy calculation of complexes.**

| **Ligands name** | Δ**Gbind** | Δ**GLipo** | Δ**Gbind vdW** | Δ**Gbind Columb** | Δ**Gbind solv GB** | Δ**Gbind Covalent** |
| --- | --- | --- | --- | --- | --- | --- |
| **Silibinin** | -124.55 | **-45.579960** | -75.91665 | **-34.697** | **30.236505** | **536.89303** |
| **Curcumin** | **-62.637497** | -22.72451 | **-47.0814** | **-23.037680** | 37.489515 | **0.033819** |
| **Demethoxycurcumin** | -39.111 | -5.8760 | -13.9152 | -15. 7191 | 19.5368 | 0.6443 |
