# Supplementary material for: Silibinins and curcumin as promising ligands against mutant cystic fibrosis transmembrane regulator protein
Source: AMB Express. 2024 Jul 23;14:84. doi: 10.1186/s13568-024-01742-z (PMC11266341; doi:10.1186/s13568-024-01742-z)
Supplement: Supplementary file 2 — Supplementary Material 2. [file 13568_2024_1742_MOESM2_ESM.docx]

**Supplementary Material Figures**


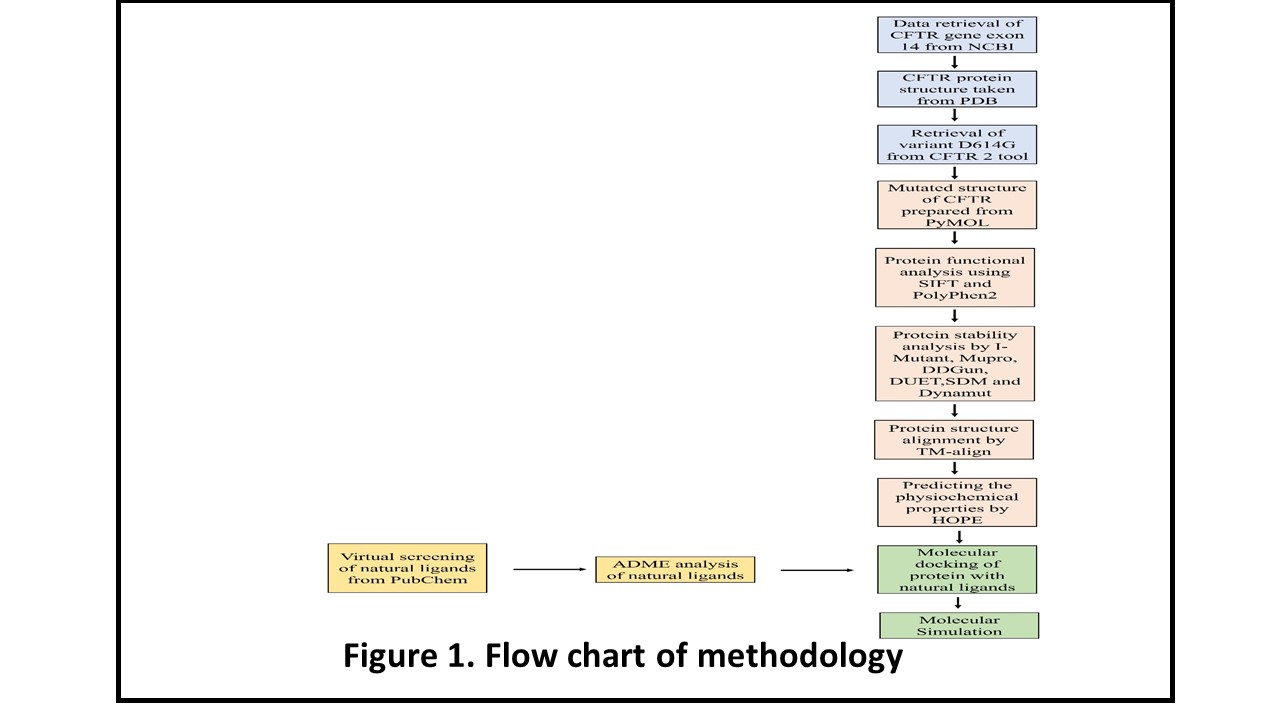


**Supplementary Fig 1**. Flowchart of methodology


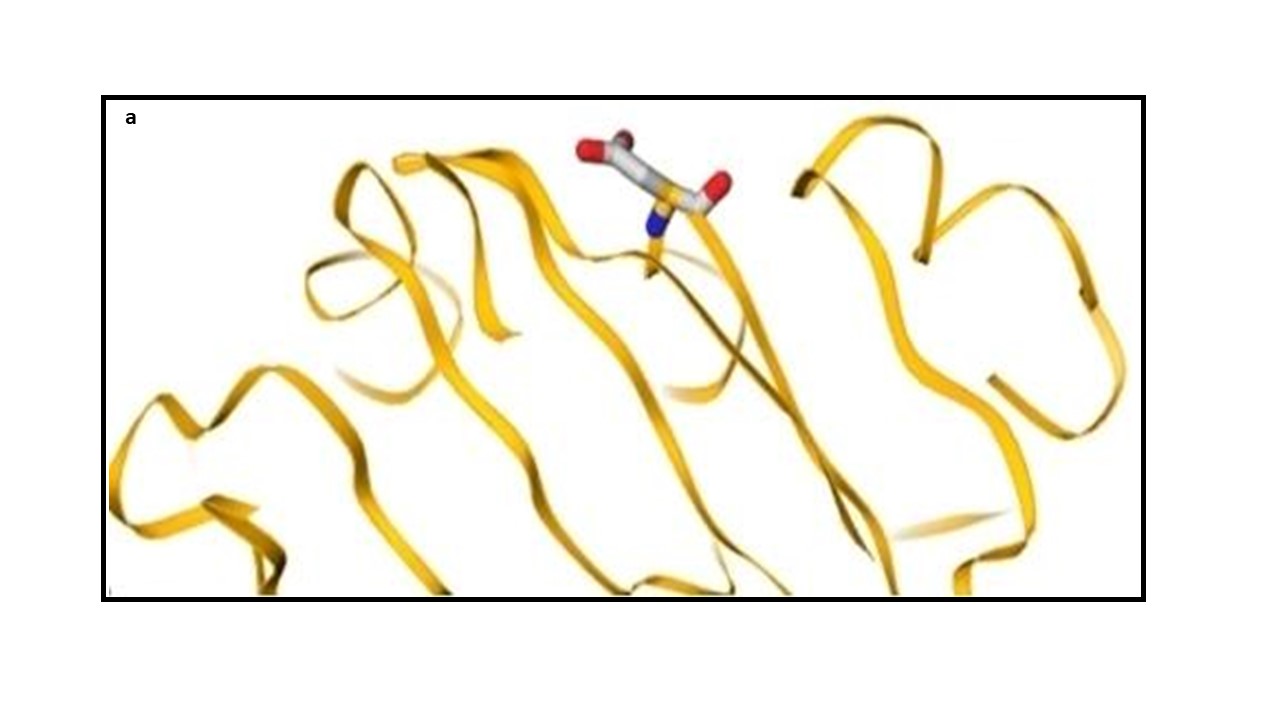


**Supplementary Fig 2**. Structure of mutated CFTR protein, with the mutated residue (G) represented as atomic structure.


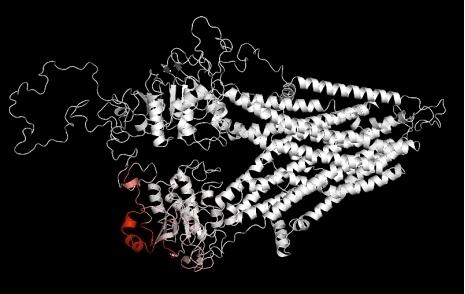


**Supplementary Figure 3.** Visual representation of vibrational entropy energy. The mutation results in the coloration of amino acids changing in accordance with the vibrational entropy. Flexibility is increased when the color is red.


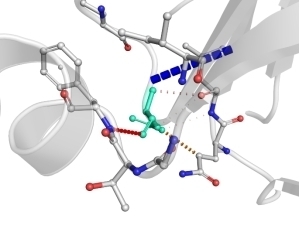

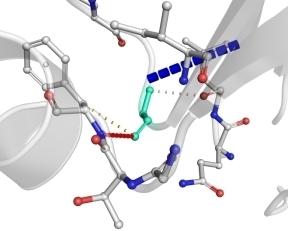


**Supplementary Fig 4. (a&b)** Interatomic interactions of wild type and mutated proteins on site of mutation. The surrounding residues that are interacting in any form of are depicted as sticks alongside the wild type and mutant residues, which are both colored light green. The blue and red color represents halogen bonds and hydrogen bonds respectively.


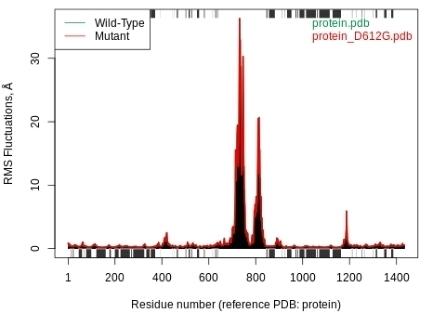


**Supplementary figure 5.** Normal mode analysis of wild type (green) and mutated (red) CFTR. The top and bottom edges of the figure are added with the secondary structure type for each area of the sequence (helices are black and strands are grey).


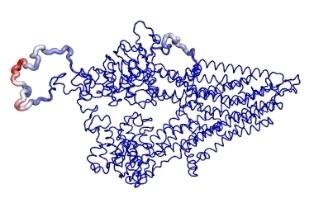
**(a)**
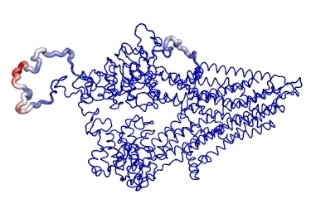


**(b)**

**Figure 6.** Atomic fluctuations of wild type (a) and mutant (b) CFTR protein. The magnitude of the fluctuation is represented by thin to thick tubes colored blue (low), white (moderate), and red (high).


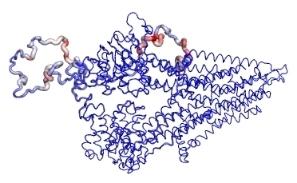


(a)


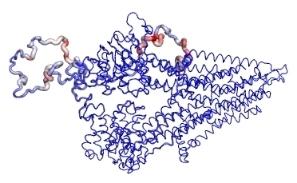


(b)

**Supplementary figure 7.** Visualization showing the deformation energies of naturally occurring (a) and mutant (b) CFTR. Thin to thick tubes in the colors blue (low), white (mid), and red (high) are used to depict the deformation's degree of distortion (high).


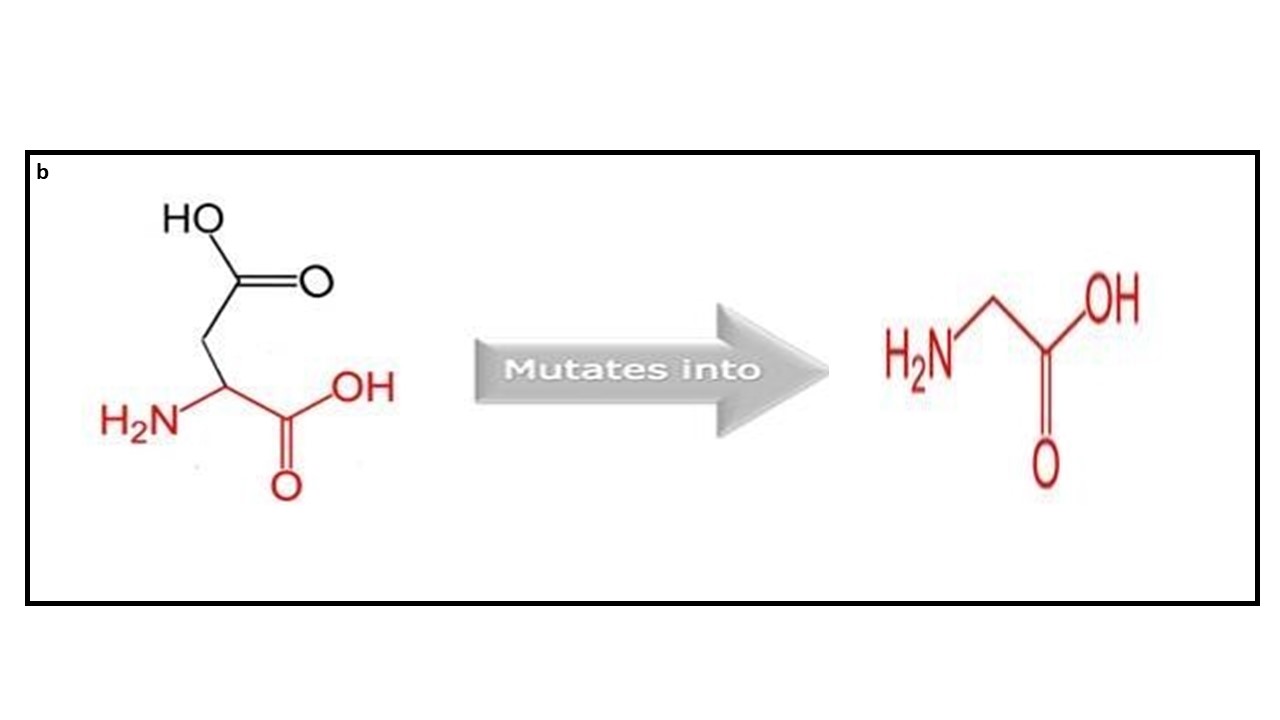


**Supplementary Fig 8.** shows the schematic representation of the original (left) and the mutant (right) amino acid. The backbone is the same for, but the side chain is different as shown in black. The mutant residue is smaller and more hydrophobic as compared to the wild one. The mutant residue Glycine is very flexible and disturbs the required flexibility of the protein.


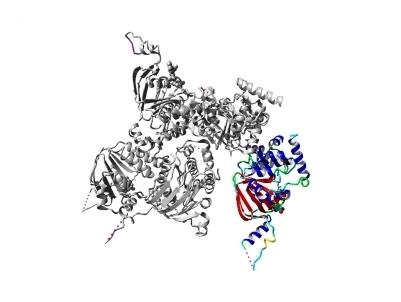


**Supplementary Fig 9.** **CFTR** Protein in ribbon presentation. The protein is colored by the element alpha helix=blue, beta strand=red, random coil=cyan. Others are shown in grey.


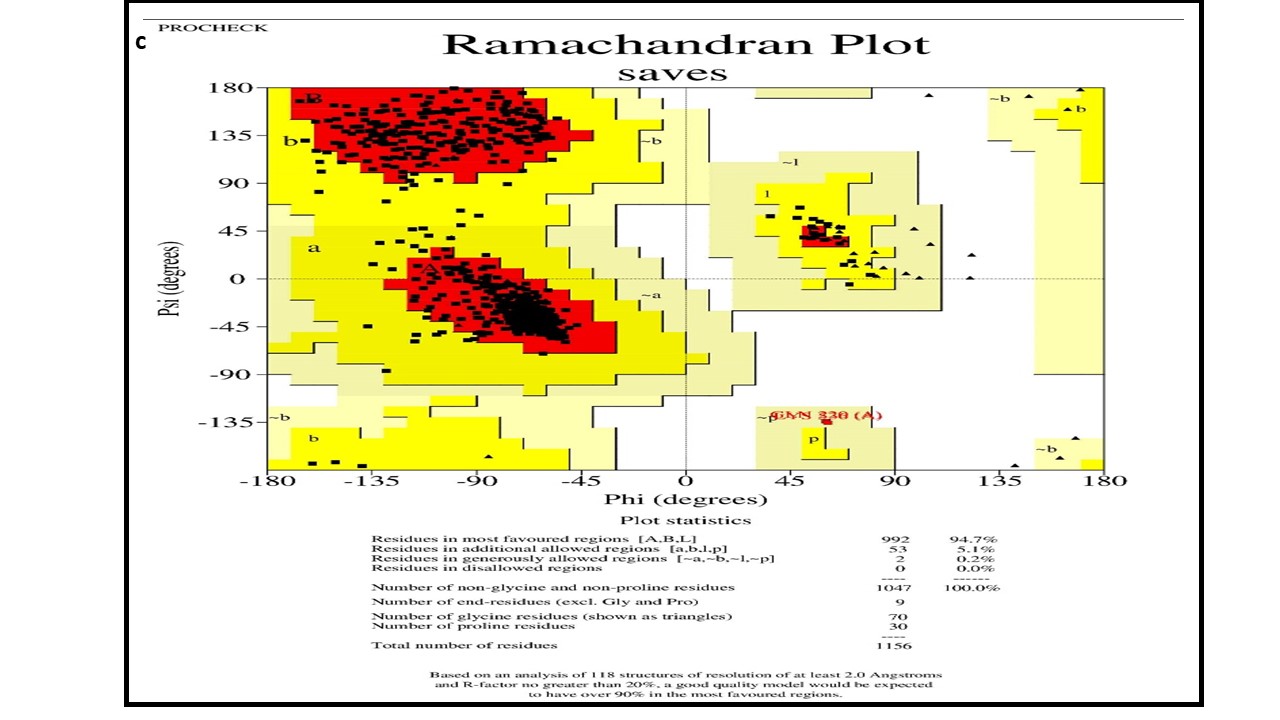


**Supplementary Fig 10.** Ramachandran plot of CFTR protein structures. (a) Ramachandran plot of mutated CFTR protein (D614G).

**
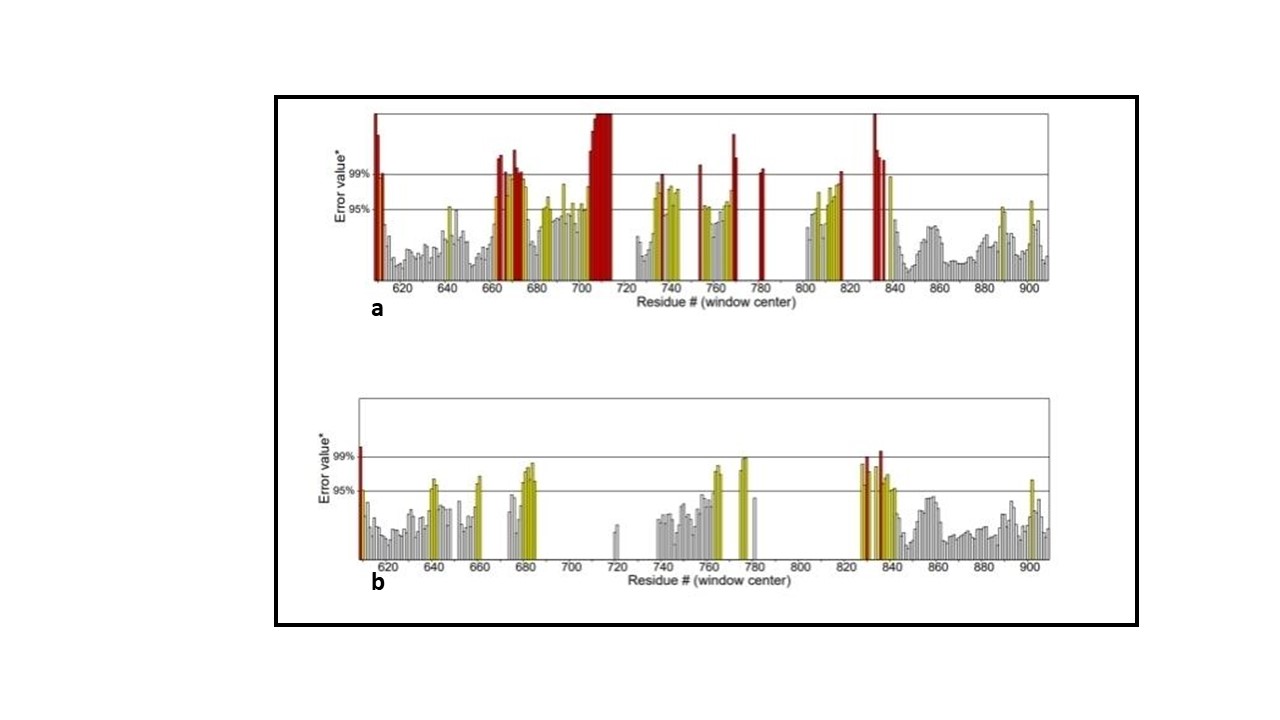
 Supplementary Fig 11.** Residue wise predicted ERRAT graphs of standard (a) and mutated (b) CFTR. The window center represents the residue subjected to mutation.

**
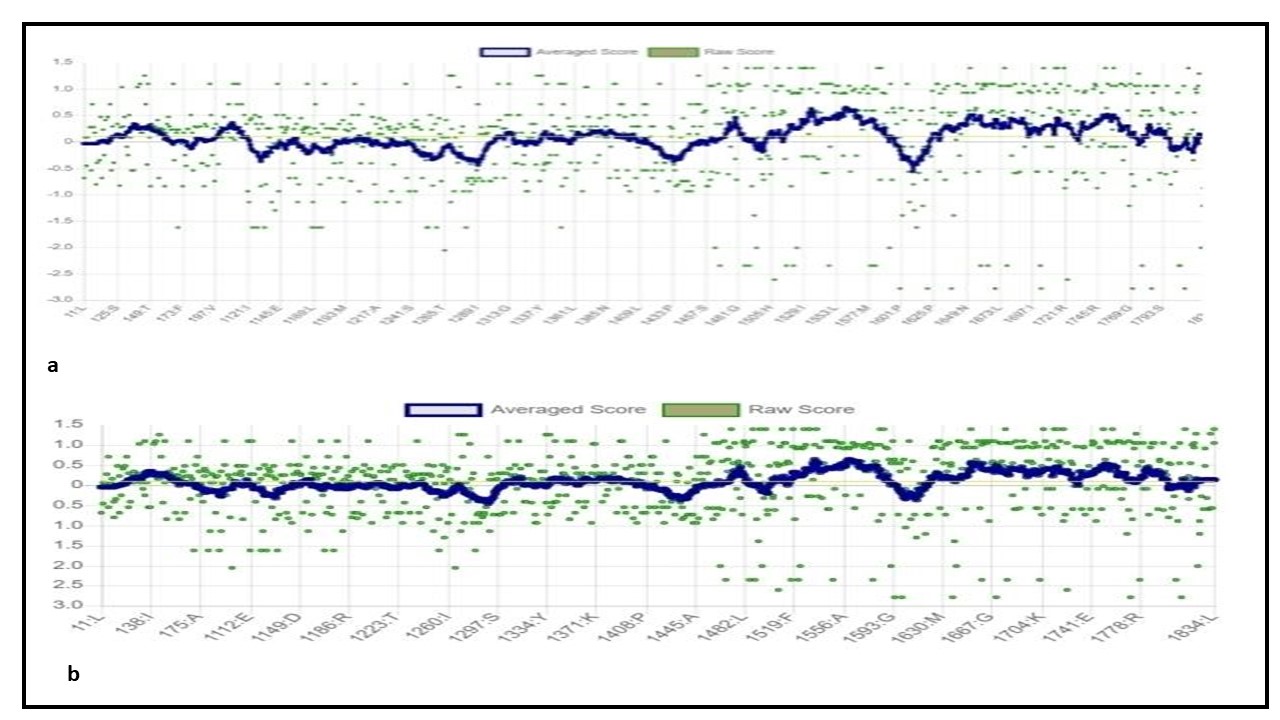
 Supplementary Fig 12.** Verify3D graphs representing average and raw scores of normal (a) and mutated (b) CFTR proteins.


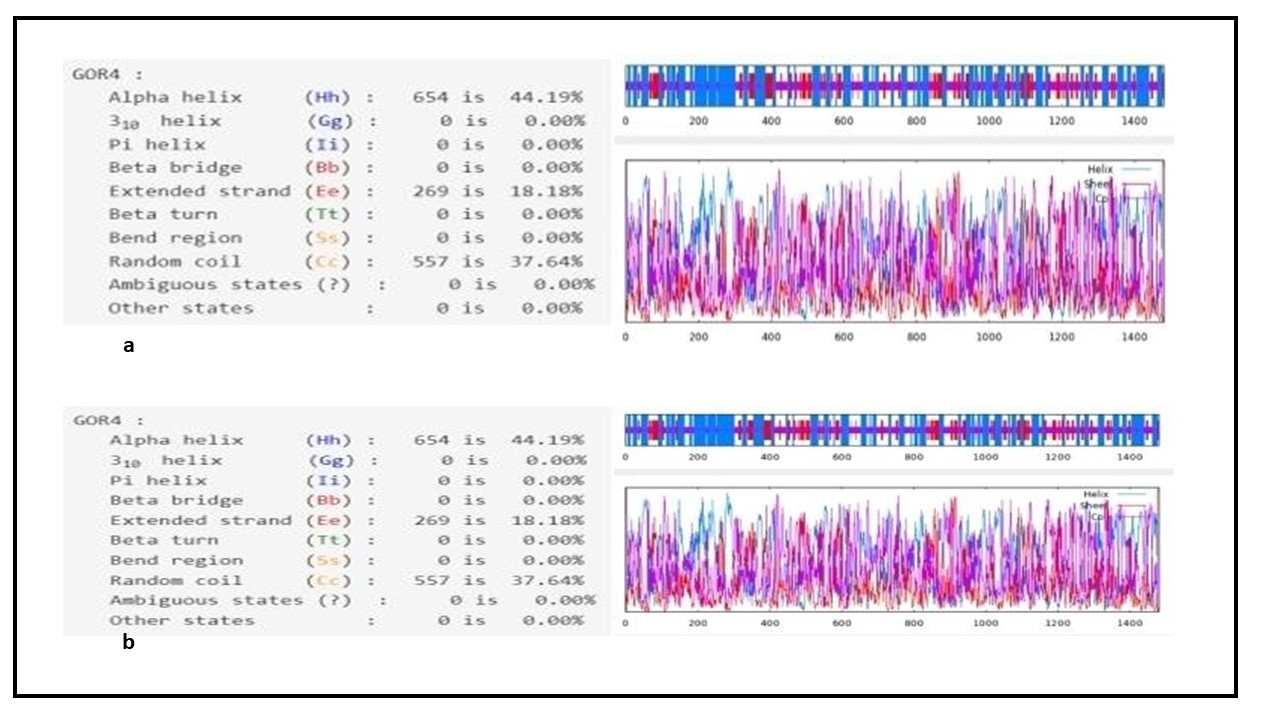


**Supplementary Fig 13.** The secondary structure of mutated CFTR protein was predicted using the GOR IV method, displayed as an alpha helix (blue), extended strands/ sheets (red), and random coils (purple).


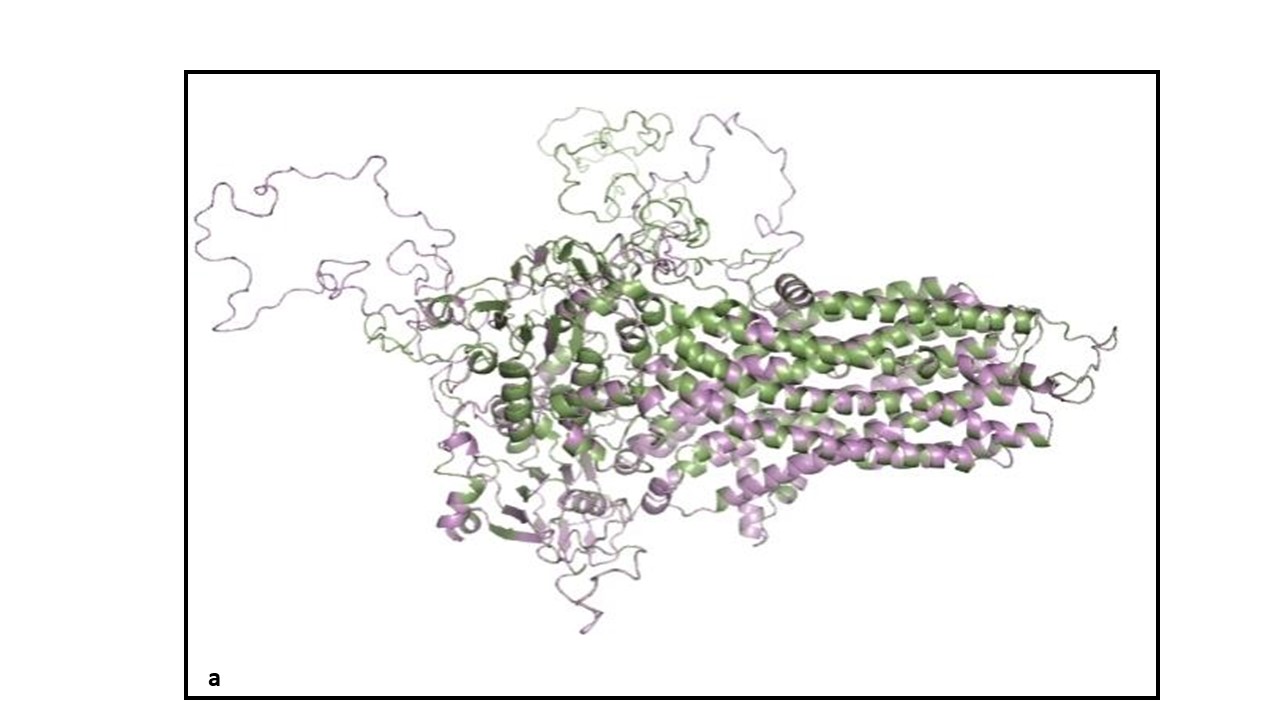


**Supplementary Fig 1)**. Superimposed structural display of normal CFTR (lavender) and mutated CFTR (green).


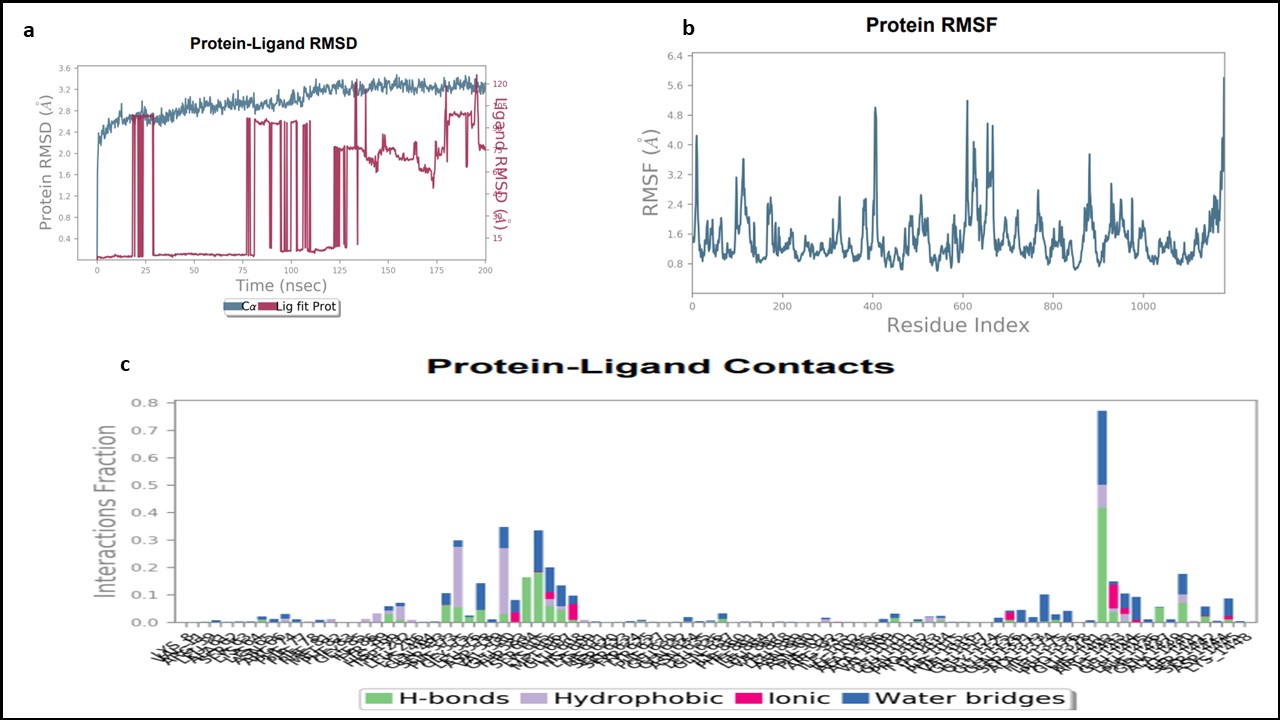


**Supplementary fig 15: MD simulation analysis of the Demethoxycurcumin with CFTR** (D614G) **complex.** (a) RMSD of CFTR protein. (b) The protein RMSFs represent the individual fluctuations of each amino acid during the simulation. (c) The protein-ligand contact histogram displays the distribution of contact between the protein and the ligand.
